# A decentralized blockchain-based smart framework for continuous vehicle emission monitoring in smart cities

**DOI:** 10.1038/s41598-025-22925-z

**Published:** 2025-11-10

**Authors:** J. Chandra Priya, Dhinesh Balasubramanian, R. Nikil Sri Shen, Choo Wou Onn, Utku Kale, Jonas Matijošius

**Affiliations:** 1 Department of Computer Science and Engineering, AAA College of Engineering and Technology, Virudhunagar, Tamil Nadu India; 2 Department of Electronic Engineering, Research Institute of IoT Cybersecurity, National Kaohsiung University of Science and Technology, Kaohsiung, Taiwan; 3 Department of Mechanical Engineering, Mepco Schlenk Engineering College, Sivakasi, Tamil Nadu India; 4 Department of Port Engineering, Lithuanian Maritime Academy (LMA), Vilnius Gediminas Technical University, Klaipėda, Lithuania; 5 Independent Researcher, Madurai, Tamil Nadu India; 6 Faculty of Data Science and Information Technology, INTI International University, Nilai, 71800 Negeri Sembilan Malaysia; 7 Department of Aeronautics and Naval Architecture, Faculty of Transportation Engineering and Vehicle Engineering, Budapest University of Technology and Economics, Műegyetem rkp. 3, Budapest, H-1111 Hungary; 8 Mechanical Science Institute, Vilnius Gediminas Technical University, Plytinės g. 25, Vilnius, LT- 10105 Lithuania

**Keywords:** Blockchain, Internet of things, Pollution control, Smart system, Smart city, Vehicle emission monitoring, Health care, Engineering, Mathematics and computing

## Abstract

Due to its high emissions, vehicular air pollution remains a critical contributor to environmental degradation and global warming; however, even in smart cities, control mechanisms often remain inadequate. The current Pollution Under Control Certificate (PUCC) system suffers from inefficiencies such as weak monitoring, maintenance gaps, and data manipulation risks. This paper proposes a blockchain-enabled framework integrating Internet of Things (IoT) sensors, machine learning (ML), and decentralized data validation to enhance emission control. In the proposed system, IoT-based sensors installed in vehicles continuously monitor emission levels and transmit real-time data to a blockchain network, ensuring tamper-proof, transparent, and immutable records. A consortium blockchain is used to validate and store emission data across distributed nodes. Furthermore, the eXtreme Gradient Boosting (XGBoost) machine learning model is applied to this data to predict emission trends and identify vehicles requiring maintenance proactively. Comparative simulations with cloud and fog-based models demonstrate the system’s superiority: the blockchain-based XGBoost model achieved 97.98% prediction accuracy, outperforming cloud systems by 4.9%. Additionally, the proposed system delivered a throughput of 679 Mbps, the response time of 91.98 milliseconds, and a processing time of 225.88 milliseconds. This framework overcomes PUCC system limitations, offering a scalable and reliable approach for reducing vehicular pollution in support of smart cities and sustainable urban development.

## Introduction

Global warming, driven by greenhouse gases, is a major challenge, causing rising sea levels and extreme weather. The increased vehicle use significantly contributes to air pollution and climate change through the emission of particulate matter, CO2, nitrogen oxides, and chlorofluorocarbons. According to research, these emissions contribute significantly to respiratory, cardiovascular, and even cancer conditions^1,2^. The association between outdoor air pollution, notably particulate matter, and lung cancer, as discussed in^3^, emphasizes the urgent need for effective mitigation strategies and public health initiatives. Addressing vehicular air pollution requires a comprehensive, multidisciplinary approach. Global advancements in air pollution control^4^ with an emphasis on pollutant detection are highlighted in a systematic review (1998–2022). According to a year-long study conducted in Bengaluru^5^, tracking CO2, black carbon, and ultrafine particles revealed that highway pollution surpasses residential streets, emphasizing advanced monitoring for improved urban air quality. Despite Bharat Stage Standards, cleaner fuels, and the Pollution Under Control Certificate (PUCC) system, emission control faces challenges due to regulatory deviations, transparency issues, and a lack of auditing, reducing PUCC effectiveness. Beyond vehicles^6^, environmental initiatives significantly impact organizational performance. A study on Vietnamese Small and Medium-sized Enterprises^7^ showed that while environmental measures improve performance, overinvestment in pollution control can also negatively impact. This highlights the need for a balanced approach to environmental investments, ensuring sustainability without compromising economic efficiency.

According to the study^8^, the existing PUCC system has inefficiencies and has not improved much despite an increase in the number of vehicles. We provide a blockchain-based system that decentralizes monitoring while ensuring data immutability, security, and transparency. The peer-to-peer networking among PUCC stations enhances jurisdiction-wide monitoring. By automating emission tracking, the integration of the Internet of Things (IoT) minimizes the likelihood of human error and manipulation^9^. The prior systems typically rely on centralized or monolithic architectures, which create performance bottlenecks such as network congestion, high latency, and single points of failure. These limitations inhibit the scalability, responsiveness, and real-time processing capabilities required for dynamic IoT environments. The existing blockchain-IoT solutions for emission monitoring improve data integrity and automation but face major limitations. They rely on monolithic or globally validated architectures that struggle to scale with growing vehicle numbers and emission centers, leading to network congestion and performance bottlenecks. These systems also lack mechanisms for incentivizing compliance or penalizing malpractice, failing to encourage honest participation. Also, their centralized validation models are poorly suited to diverse local regulatory contexts, increasing the risk of single points of failure, reduced contextual accuracy, and vulnerability to manipulation or collusion.

Using eXtreme Gradient Boosting (XGBoost) for predictive analytics, preventive interventions can be enabled by forecasting pollution trends. By providing timely maintenance notifications and regulatory actions, this capability reduces vehicle emissions. Because of its adaptability, the framework can be modified to suit the evolving needs of law enforcement, legislators, and vehicle owners. The technology enhances pollution monitoring, encourages sustainable urban planning, and contributes to environmental and smart city initiatives by resolving PUCC shortcomings. All symbols with descriptions are listed in Table 1.

**Table 1:** Symbols and description.

Symbol	Description
PUCC	Pollution under control certificate
XGBoost	eXtreme gradient boosting
SHA-256	Secure hash algorithm with 256 message digest
Vehicle_details_	Vehicle information set {fuel type, engine type, car model}
F_type_	Fuel type (petrol, diesel, CNG, etc.)
E_type_	Engine type
M_car_	Car model
S_set_	Sensor set installed in the vehicle
S_CO_	Carbon monoxide (CO) sensor
S_NOx_	Nitrogen oxides (NO_x_) sensor
S_HC_	Hydrocarbon (HC) sensor
S_PM_	Particulate matter (PM) sensor
MQ7, MiCS5524, TGS2600	Sensor models for detecting CO, NO_x_, and HC
PM 5503	Sensor model for detecting PM emissions
OBU_device_	On-board unit device
ESU_device_	Emission sense unit
H_ψ_	256 bit hash value of vehicle details and sensor set
T	Set of sampling time intervals
S_init_	ESU initialization status
EdgeNode_conn_	Edge node connection
TCH_hub_	Trusted consortium hub
D_emission_	Emission data collected from sensors
λ_limit_	Emission threshold limit for compliance
SC_check_	Smart contract compliance check
R_access_	Certification request by vehicle owner
MVI_auth_	Motor vehicle inspector (MVI) authorization
ElG_sign_	ElGamal digital signature algorithm
D’_emission_	Processed emission data
^Enc^D_emission_	Encrypted emission data
H^’^_ψ_	Hash of encrypted emission data
Res^*^_ψ_	Compliance result stored on blockchain
σ	Digital signature generated using ElGamal
Cert^+^_ψ_	Digitally signed PUC certificate
ElG_PK_	Public key for verifying the signature
λ_TH_	Threshold limit for emission verification
I_σ_	Integrity verification result
S_Q_	Success status of the PUC Endorsement
S_integrity_	Final verification integrity result

This study is driven by the critical need to enhance vehicular emission monitoring, where existing regulatory mechanisms, particularly the PUCC system, exhibit significant limitations in terms of transparency, auditing, and operational efficiency. The rapid growth in vehicle ownership, coupled with inadequate enforcement and outdated monitoring infrastructure, worsens air pollution and poses severe public health risks. These challenges highlight a clear gap in the current emission control framework. To address the limitations of existing vehicle emission monitoring systems, this study proposes a comprehensive blockchain- and IoT-enabled framework supported by machine learning for emission trend analysis. The key innovations of this work include a decentralized and tamper-proof data management system, real-time emission tracking, and predictive analytics to enable timely interventions. This section has established the serious need for a more transparent, scalable, and accountable vehicular emission monitoring system. This highlights limitations of PUCC mechanisms and blockchain-IoT hybrids, especially in scalability, incentives, and validation. The next section reviews related works to contextualize the gaps addressed by our approach.

To explicitly highlight our contributions, Table 2 contrasts the novel aspects of our framework against existing systems. Unlike traditional approaches, we introduce: (1) localized, role-based validation by Motor Vehicle Inspectors (MVIs) to enforce region-specific compliance; (2) token-based incentives to reward/penalize users; (3) edge computing via smartphones for scalable data processing; and (4) XGBoost-powered predictive analytics on blockchain-verified data. These novel methodologies collectively overcome the limitations of centralized validation, incentive misalignment, and reactive monitoring in current emission control systems.

**Table 2:** Contributions of the proposed framework versus existing systems.

Feature	Proposed framework	Existing systems	Novelty & impact
Validation model	Role-based validation by MVIs with jurisdiction-specific thresholds	Global/static validators	⎫ Localized regulatory compliance ⎫ Eliminates single points of failure
Incentive mechanism	Token rewards (fuel/insurance discounts) for compliance + penalties via smart contracts	No incentives	⎫ Resolves misalignment in PUCC systems ⎫ Encourages honest participation
Scalability approach	Smartphones as edge nodes + Consortium blockchain	Centralized clouds or monolithic chains	⎫ Achieves 679 Mbps throughput (4–7× higher) ⎫ Enables city-wide deployment
Predictive intervention	XGBoost forecasting on blockchain-verified emission data	Threshold-based reactive alerts	⎫ 97.98% prediction accuracy (+ 4.9% vs. cloud) ⎫ Enables proactive maintenance

The rest of the paper is organized with Sect. 2 reviewing the relevant works. Section 3 details the methodology employed in this work. An analytical model of the system is described in Sect. 4. Section 5 presents the applied implementation, followed by the experimental setup and evaluation in Sect. 6. The results and discussion are provided in Sect. 7. Finally, this paper concludes with a conclusion and future work in Sect. 8.

In summary, this study addresses the critical shortcomings of the current PUCC system by proposing a scalable, transparent, and accountable framework that uses blockchain for secure data management, IoT for real-time emission tracking, and machine learning for predictive analytics. Unlike existing systems, our approach resolves issues of centralized validation, poor scalability, and lack of incentives for compliance. By aligning technological innovation with regulatory and environmental goals, this framework sets the stage for a more effective and future-ready emission control system. The following section reviews related work to contextualize these contributions and highlight the specific research gaps our framework addresses.

## Related works

This section reviews research on the existing PUCC system, blockchain-based pollution monitoring and prediction systems, fog and cloud computing infrastructure. The presently operating PUC system^10^ has issues, such as low equipment maintenance, inexperienced operators, and inadequate infrastructure at emission centres, which results in erroneous test findings. The system also has issues with inefficient on-road inspections, a lack of centralized vehicle registration databases, an uneven distribution of centres, weak database management, and a failure to record failed vehicle data. The validity, efficiency, and equity of the pollution certification procedure are all compromised by these problems.

An IoT- and blockchain-based real-time air pollution measurement platform using 5G addressed data falsification; however, it faced challenges in large-scale industrial deployment^11^. Several approaches have explored the secure storage of pollutant data using blockchain platforms^12^; however, these often encounter challenges such as long execution times and significant overhead in node configuration. Other solutions have aimed to automate processes like permit trading and carbon emission audits^13^, still they frequently face scalability issues due to lengthy setup and execution times. Most existing blockchain-IoT frameworks rely on either global or static validators that operate independently of local regulatory environments, thereby limiting their capacity to enforce region-specific compliance requirements. Another work^14^ predicts PM2.5 using IoT and machine learning (ML); however, cloud dependence raises latency in places with limited connectivity. While some prior models incorporate basic data analytics or threshold-based alerting, they generally lack advanced, proactive intervention mechanisms capable of autonomously responding to anomalous patterns or critical events in real time, thereby limiting system adaptability and resilience. Vehicle pollution was measured in Beijing using a real-time traffic analysis^15^, which suggested emission reduction scenarios that might reduce pollutants by up to 21%. However, more reductions are required to achieve significant enhancements in air quality. Though sustainable development needs to be improved, the SIoT-based Peer-to-Peer (P2P) cross-ledgering architecture^16^ improves blockchain-driven cognitive sustainability. A consortium blockchain^17^ enhances decentralized knowledge bases; however, complexity in governance and vandalism continue to be major challenges.

Instead of using XGBoost, the study^18^ employed a GA-LSTM model for air pollution prediction to improve accuracy; nevertheless, this model had computational inefficiencies. In an IoT-based air pollution prediction system^19^, DLMNN and H-ANFIS indicated enhanced efficiency, although they were not as fast or scalable as XGBoost. With large-scale real-time data, a wavelet-LSTM model^20^ improved emission estimates, although it had issues. Macau SAR’s multi-scale pollution model^21^ effectively depicted pollution patterns, although it underestimates PM concentrations. While it performed better than standard models, the GRU-ED approach^22^ for PM2.5 prediction in New Delhi was more computationally expensive than XGBoost. The research^23^ used mass-balance principles with machine learning to analyze real-time traffic pollution; nonetheless, it experienced issues controlling reactive species and complex chemical interactions. Although the final prediction, a CFD-based BPNN model^24^, accurately estimated the dispersion of pollutants, it was challenging to generalize to complex urban situations due to its limited spatial and temporal adaptability.

To improve computational efficiency and data security in smart vehicles, the research study^25^ analyses the inclusion of blockchain and edge computing in IoT. Vehicular fog computing (VFC)^26^ reduces latency and cloud congestion by employing automobiles as fog nodes for real-time traffic control. An authentication system^27^ for drone-assisted IoV ensures safe communication and effective congestion management in high-density situations. To improve resource management and real-time data processing, the study^28^ integrates Edge Computing in IoV to optimize energy efficiency in 5G and 6G networks.

To conclude, this review demonstrates that while previous blockchain- and IoT-based emission monitoring systems have made progress in data integrity and automation, they still fall short in large-scale deployment, incentive compatibility, and efficient, context-aware validation. These persistent gaps underscore the necessity for a new framework that can scale efficiently, motivate honest participation, and enable localized, trustworthy validation. Based on this review, the key limitations and research gaps are summarized in Table 3. The next section articulates the specific problem statement that our proposed solution aims to address.

**Table 3:** Summary of research gaps in existing emission monitoring and prediction systems.

Existing works	Validation	ML/DL prediction	Focus area	Challenges addressed	Technologies employed	Key limitations & research gaps
^8^	✗	✗	Pollution control system	Suboptimal performance of current pollution control systems	Infrastructure assessment, calibration, maintenance	No decentralized oversight, corruption vulnerability, non-compliance, awareness gap, limited optimization
^9^	✗	✗	Industrial air pollution monitoring	Ineffective monitoring for industrial pollution	Blockchain, 5G	Limited scope (industrial focus), cost inefficiency, compliance neglect
^11^	✗	✗	Automated carbon emission auditing	Insecure emission data distribution and audit	Hyperledger fabric, IoT, blockchain	Government oversight, data security, no predictive capabilities
^14^	✓	✗	Secure data exchange	Secure information sharing among stakeholders	Blockchain, P2P Cross-Ledgering	Centralized oversight, no owner engagement
^17^	✗	✗	Air pollution prediction	Sensor faults, faulty data in IoT networks	H-ANFIS, MPCA, DLMNN, IoT	No validation linkage, no decentralized data integrity
^18^	✗	✗	Vehicle emission forecasting	Missing data in vehicle emission forecasting	Wavelet transform, lstm, semi-supervised regression	Limited scope (forecasting only), no fraud prevention
^19^	✗	✗	Air pollution forecasting	Inaccurate prediction of air pollutants	GRU-ED	Data discrepancies, no compliance enforcement, oversight deficiencies

### Problem statement

The efficiency of the PUCC system to reduce vehicle emissions is compromised by serious operational and structural shortcomings. Since many centres issue certifications without conducting adequate inspections, corruption and fraud are pervasive with fabricated compliance records. The system is further weakened by inadequate testing procedures, since tests for petrol vehicles do not integrate with onboard diagnostics (OBD), and tests for diesel vehicles only measure smoke density, excluding important pollutants like NOx and particulate matter. These problems are made worse by weak enforcement and control, which allows decentralized centres to function with no oversight, resulting in anomalies and ineffective penalties. Software bugs, GPS tracking errors, and persistent manual data entry are types of technical and operational shortcomings that lead to inconsistencies in emissions databases, which impede compliance efforts. Additionally, misaligned incentives encourage malpractice, as operators prioritize volume over accuracy due to flat testing fees, while vehicle owners seek non-compliant centers to bypass retesting. There is a need for a decentralized consortia integrating transparency, real-time monitoring, and predictive analytics for proactive emission control.

The system comprises entities such as V (vehicles), C (PUCC centers), T (testers), O (owners), and G (government), aiming to maximize owner awareness and compliance (Σ_v_ (aₒ·cₒ)) while minimizing government monitoring costs (Cg(V)). This involves increasing infrastructure/equipment quality (i_c_), tester expertise (e_c_), and oversight (o_c_) at each center C, improving owner awareness (aₒ) and compliance (cₒ) for each owner O, and implementing decentralized monitoring. The key constraints include M_v_(c) = E_v_(c) + ε(i_c_, e_c_), where M_v_(c) is measured emission, E_v_(c) is actual emission, and ε represents the measurement error; P_v_(c) = 1 − o_c_·aₒ·cₒ, where P_v_(c) denotes the probability of fraudulent certificate issuance; and Cg(V) ∝ Σ_c_(1 − i_c_·e_c_·o_c_)·|V|, representing the government monitoring cost. The challenges are to improve measurement accuracy (M_v_,ₜ ≈ E_v_,ₜ), prevent fraudulent certifications (C_v_,ₜ = 1 ⇨ M_v_,ₜ ≤ τ), maximize compliance (C_v_,ₜ = 1 when E_v_,ₜ ≤ τ), and enforce effective oversight (O_c_,ₜ = 1 ∀c, t), where E_v_,ₜ is actual emission, M_v_,ₜ is measured emission, C_v_,ₜ is certificate status, A_v_,ₜ is owner awareness, O_c_,ₜ is center oversight at time *t*, and τ is the emission threshold. As detailed in Table 4, prior approaches suffered from key limitations such as high latency, lack of decentralization, and weak predictive capabilities.

**Table 4:** Quantitative comparison of metrics across state-of-the-art systems.

Ref.	Accuracy (%)	F‑Measure (%)	Throughput (Mbps)	Response time (ms)	Processing time (ms)	Limitations
^8^	-	-	~ 320	150–200	> 500	Lacks scalability
^10^	-	-	280	180	420	High setup time; no predictive analytics
^13^	92.5	89.3	450	95	300	Centralized oversight
^16^	89.7	85.1	210	220	600	High latency; relies on cloud infrastructure
^17^	91.2	87.6	-	130	350	No fraud prevention
^18^	88.4	83.9	-	-	-	No compliance enforcement
^19^	93.1	90.5	-	110	410	Computationally expensive GRU‑ED model

### Our contribution

This paper proposes a blockchain-enabled PUCC framework to enhance transparency, security, and accountability in emission monitoring using IoT devices. It eliminates reliance on third-party centers, reducing manipulation and inaccuracies. The machine learning model analyzes emission data to predict trends, enabling timely alerts and proactive compliance to ensure data reliability, cost-effectiveness, and improved environmental impact. The primary objective is to design a novel framework that establishes a network among test centers and imparts accountability among vehicle owners. To achieve this, the framework includes the following contributions:

- A transparent, secure, and highly scalable blockchain framework that ensures secure storage of readings and enables efficient monitoring and tracking of users.
- Our framework proposes a consortium blockchain integrated with edge computing capabilities through user smartphones. These personal mobile devices act as edge nodes for preliminary IoT sensor data processing, enabling distributed load handling and significantly enhancing system scalability as the number of vehicles increases. This reduces dependency on centralized cloud infrastructure and ensures cost-effective horizontal scaling, making it viable for large-scale urban deployment.
- A robust validation framework for issuing endorsements by members of peer-to-peer networks, conducted by trusted entities known as Motor Vehicle Inspectors (MVI).
- An IoT-based framework for user-friendly emission reading, eliminating reliance on trustless intermediaries within the system.
- A machine learning-based framework for predicting future emission trends, promoting accountability among users.

The proposed framework introduces several key innovations that distinguish it from prior blockchain-IoT emission monitoring systems:

- A multi-layered consortium blockchain designed for jurisdiction-wide deployment enables seamless scaling across regions, minimizes network congestion, and supports efficient peer-to-peer validation by local authorities like MVIs.
- It embeds incentives for vehicle owners, rewarding honest reporting and penalizing fraud, and resolves the incentive misalignment in the current PUCC and earlier blockchain systems.
- It enables local MVIs to validate and endorse emission certificates within their jurisdictions under region-specific standards and regulatory thresholds, thereby ensuring context-aware data integrity. This peer-to-peer model enhances trust, reduces bottlenecks, and mitigates centralized manipulation and collusion, which are the limitations of prior global validation approaches. Hence, this role-based consensus greatly reduces the risk of single points of failure and promotes geographically distributed governance.
- The existing systems often overlook enforcement mechanisms that promote honest participation or penalize manipulation. However, the proposed framework introduces smart contracts to automate compliance tracking and facilitates incentive structures for both vehicle owners and PUCC centers.
- The system combines cost-effective sensors with XGBoost-based analytics for automated emission data capture and real-time trend forecasting, enabling timely interventions and maintenance, surpassing prior isolated IoT or ML approaches.

**Table 5:** Comparison of proposed framework with representative blockchain-IoT systems.

Feature	Proposed framework (this work)	PUCC system (current practice)	Cloud-based blockchain-IoT	Fog/edge-enabled IoT	Blockchain-IoT without incentives
Validation	Contextual and localized by MVIs (role-based)	Centralized and manual validation	Static/global validators	Distributed data aggregation, but partial validation	Global validation with no local context
Scalability	Edge-enabled via user smartphones for distributed load	Manual, paper-based, or isolated databases; not scalable	Cloud-dependent bottlenecks with city-scale data	Lack true peer-to-peer scalability or consensus	Monolithic or globally distributed; Limited protocol throughput
Intervention style	ML-driven, predictive alerts	Periodic, reactive	Threshold-based, reactive	Rule-based or time-triggered, mostly reactive	Data logging and static alerts only
Fraud resistance/incentives	Reputation-based smart contracts + incentives	Fee-based, prone to fraud	No incentive or penalty design	Integrated, trust assumed only	No fraud control

By integrating IoT automation and predictive analytics within a secure, consortium-based blockchain, our approach provides a complete solution to the multifaceted challenges of emission control as shown in Table 5. The next section presents the methodology and architecture of our proposed system.

## Methodology

**Fig. 1:**
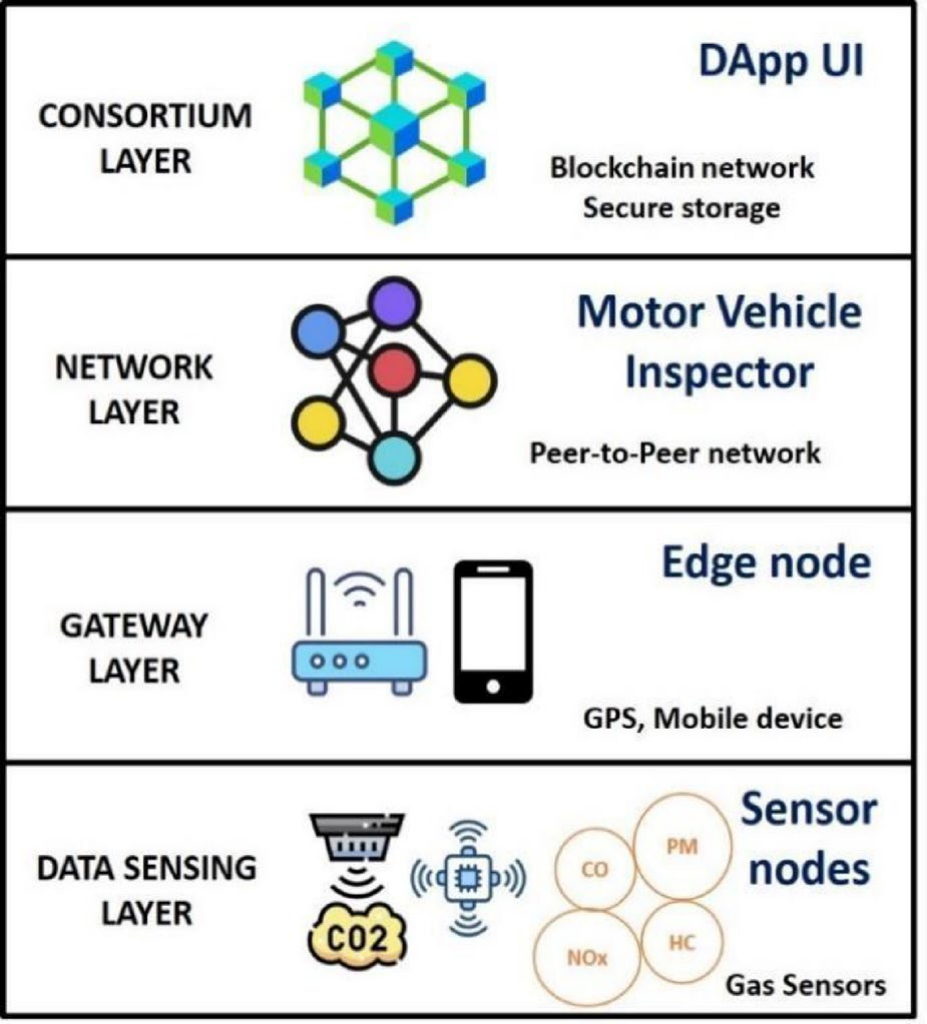
Proposed PUCC layered architecture.

After analyzing the PUCC system in the study^8^ and a Supreme Court report^27^, the problem statements were identified. The literature survey on pollution monitoring systems^9–15^ informed that blockchain-IoT supports sensor customization to detect multiple vehicle emissions. A notable novelty is its predictive capability, with XGBoost being selected as the optimal model based on reviews^16–22^ for its accuracy, efficiency, and scalability. Additionally, fog and cloud computing^23–26^ were reviewed as benchmarks, leading to NS2-based simulations for comparison and a layered architecture^33–39^. Figure 1 illustrates the proposed multi-layered decentralized application (DApp) architecture specifically designed to enhance vehicle emission monitoring and control. This layered structure is crucial for ensuring seamless data flow, real-time processing, and trustworthy emissions reporting. At the base, the Data Sensing Layer utilizes IoT-enabled gas sensors (CO₂, NOx, HC) to detect pollutant levels emitted from vehicles in real-time, enabling accurate and continuous monitoring. The Gateway Layer connects these sensors with edge devices such as mobile phones and GPS units, facilitating immediate data transfer and geolocation tagging of emissions. The Network Layer supports peer-to-peer communication among stakeholders, including vehicle owners, regulatory authorities, and emission centers, ensuring efficient dissemination and synchronization of information. At the top, the Consortium Layer provides blockchain-based storage and security, preserving the immutability and transparency of emission records while eliminating manipulation risks. The proposed architecture consists of four components: Owner Enrolment for secure registration, IoT-Enabled Data Monitoring for automated emission tracking, Trusted Consortium Hub (TCH) for decentralized transparency, and a Predictive Analytics Engine for forecasting emissions and proactive pollution control. This scalable approach effectively addresses existing system limitations.

Hence, the proposed methodology integrates a multi-layered blockchain-IoT architecture with real-time sensing, edge-based data transfer, and decentralized validation to ensure secure and scalable emission monitoring. The inclusion of a predictive analytics engine using XGBoost adds a proactive layer to emission control, addressing the limitations of current systems. This modular framework supports practical implementation and simulation. The next section presents the system design and performance.

### Owner enrolment

The owner must complete a one-time registration valid for the entire lifespan of the vehicle through a decentralized application (DApp). Each vehicle is equipped with an On-Board Unit (OBU) that segregates the vehicle into fuel type, model type, and engine type (BS-IV or BS-VI). Specifically, the OBU collects a unique electronic number V_en_​, a password V_pd_, and location information V_lc_​ from the owner. The vehicle holds a private key (PR​) and computes V_pdd_ and $\:{{\rm\:Y}}_{a}$​ which are securely forwarded to the blockchain network where a unique public address is generated for each user via the Metamask wallet. The address is derived using elliptic curve cryptography to ensure security. The users must provide their public address and passphrase for authentication, allowing access to the DApp dashboard. Within the dashboard, users can take tests, procure endorsements, and access comprehensive emission data.

Therefore, the enrolment process uses cryptographic hashing and blockchain addresses to ensure secure vehicle identity, authentication, and DApp access, leading to IoT-based real-time emission monitoring. The next section outlines the IoT-enabled data monitoring mechanism for real-time emission tracking.

**Algorithm 1:**
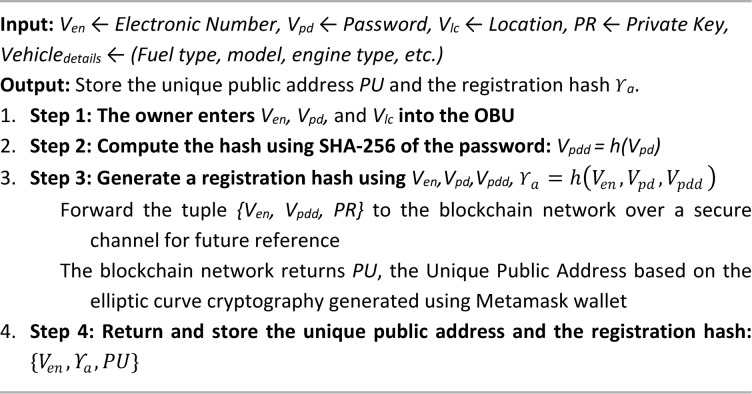
Vehicle registration

**Algorithm 2:**
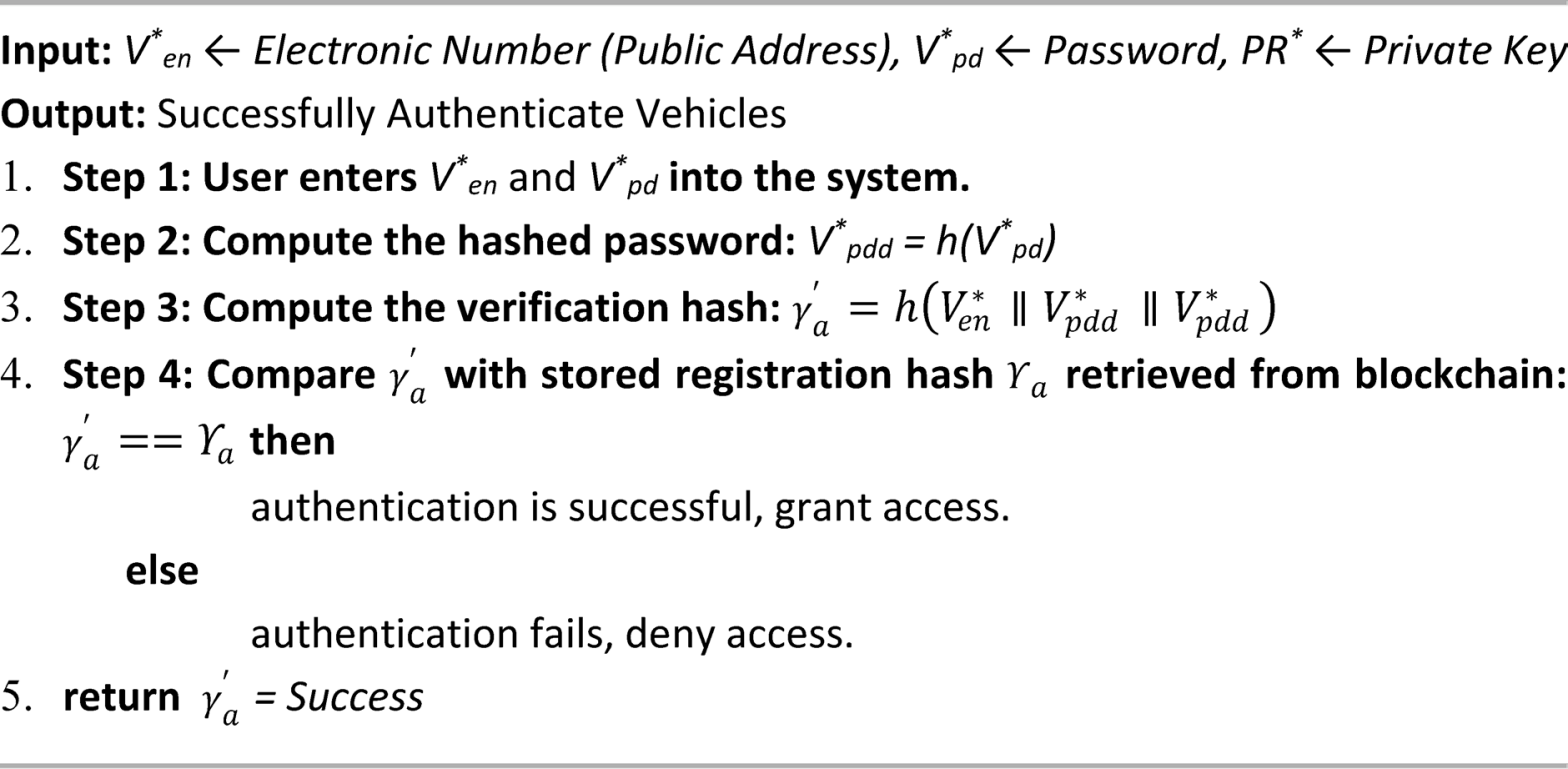
Vehicle authentication

### iot-enabled data monitoring

After owner enrolment via the OBU, vehicles are fitted with the Emission Sense Unit (ESU) to monitor emissions based on fuel type. Petrol/CNG vehicles emit CO, NOx, and HC, detected by MQ-7, MiCS-5524, and TGS2600 sensors, while Diesel vehicles emit PM2.5 (smoke density), monitored by the Plantower PMS5003 sensor.

**Fig. 2:**
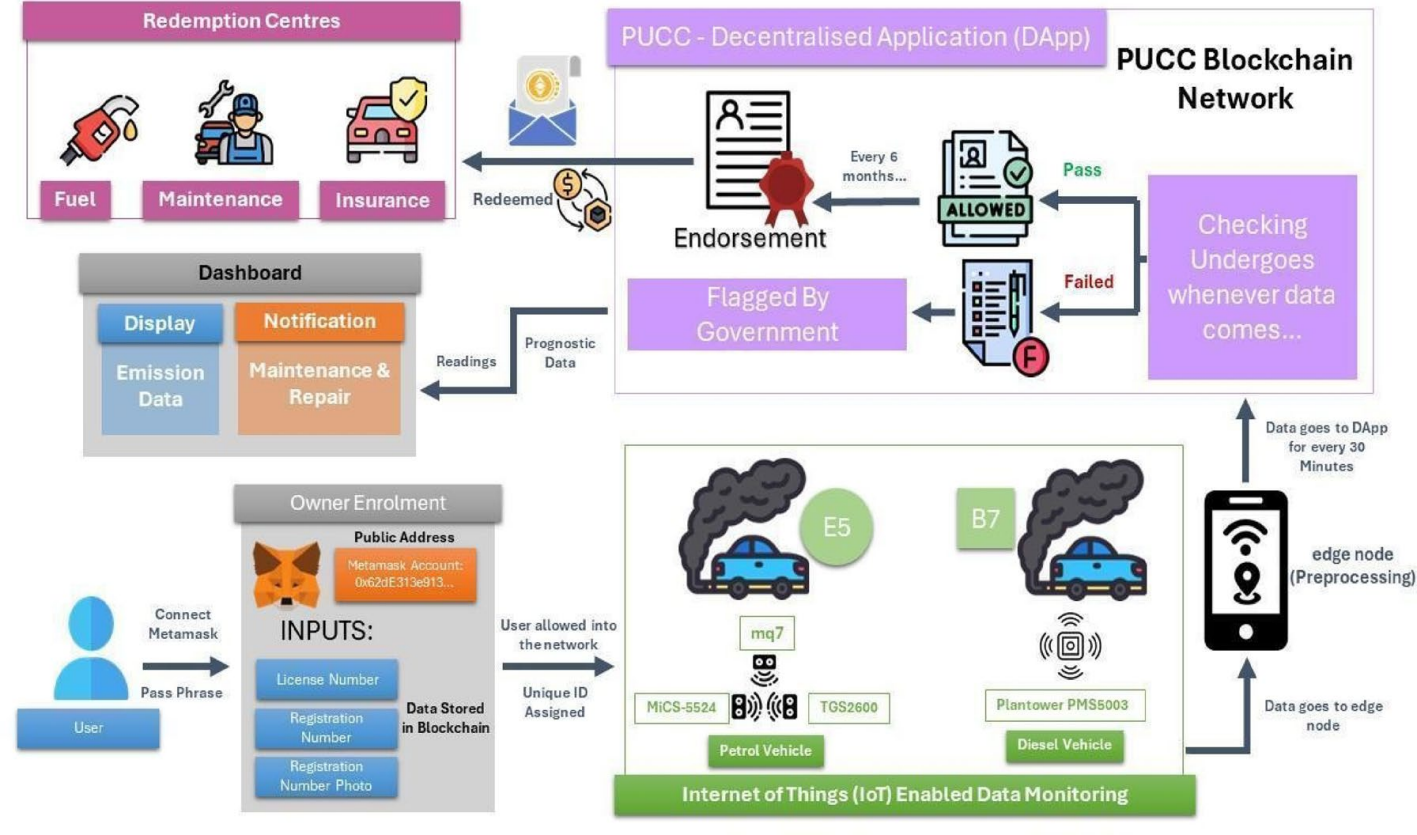
Blockchain-based vehicle emission monitoring-user side view.

These IoT sensors, installed in the vehicle’s tailpipe, enable real-time emission tracking, ensuring compliance with the proposed framework’s technical requirements. To enhance the ESU, an edge node—typically the user’s mobile device—is integrated for faster data processing, reduced latency, and efficient communication. Emission readings are updated every 15 min to both the blockchain network and the user, ensuring seamless monitoring. At the edge node, data cleaning is done, real-time emission data from IoT sensors is first filtered to remove noise and irrelevant entries, then analyzed to detect anomalies or missing values. This process ensures only accurate and meaningful data is retained. Aggregation is then performed to condense the data, minimizing transmission traffic while preserving essential emission patterns for further processing. Verified data is securely transferred to the TCH for validation and further utilized by the Predictive Analytics Engine to forecast potential emission trends, enabling proactive pollution control measures. While the MQ-7, MiCS-5524, TGS2600, and Plantower PMS5003 sensors were chosen for practicality, the framework remains scalable and adaptable, allowing future upgrades to more advanced sensors without disrupting its architecture.

The selection of sensors balances cost, scalability, and operational needs for large-scale vehicular emission monitoring. With a lower total cost per vehicle, these sensors are highly suitable for deployment in developing countries such as India and Vietnam, where affordability is critical. Their low-power operation (< 100 mA) enables seamless integration with smartphones or microcontrollers like Arduino without external power supplies. However, the alternatives such as OPC-N3 require dedicated power sources. While high-precision sensors like Alphasense provide higher accuracy, they demand frequent recalibration, making them impractical for widespread user deployment. The field validation studies, such as the use of PMS5003 in Beijing traffic and MQ-7 in Bengaluru, further support their applicability in real-world environments. Recognizing limitations such as a ± 5% error margin in CO measurement compared to ± 1% in lab-grade sensors and cross-sensitivity to gases like H₂ and LPG, the system mitigates these issues through multi-sensor fusion that flags outliers for manual review. Moreover, the modular API design allows for seamless sensor upgrades (e.g., to TGS5141 or OPC-N3) as technological and regulatory requirements evolve, ensuring long-term adaptability without architectural overhauls.

This modular approach ensures long-term sustainability and flexibility in response to evolving environmental regulations and technological advancements. By integrating real-time monitoring, predictive analysis, and blockchain security, the framework offers a comprehensive solution for modernizing vehicle emission control, improving compliance, and reducing environmental impact. Figure 2 depicts the DApp architecture from the user’s perspective. The system begins with vehicle owner enrollment, where user and vehicle data are stored on the PUCC blockchain. Emission data is periodically collected via IoT-enabled monitoring, preprocessed by an edge node, and analyzed by the DApp to determine compliance. The flagged vehicles require government endorsement, and owners receive notifications for maintenance or repairs. A dashboard provides emission insights and predictive analytics, while the DApp also enables token redemption at insurance and fuel stations.

In summary, the IoT monitoring system uses cost-effective, low-power sensors and edge preprocessing to deliver accurate, real-time emission tracking. The modular design supports adaptability to future sensor upgrades, while blockchain integration ensures secure and transparent data flow. This system enhances emission compliance and user accountability. The following section details the decentralized validation handled by the TCH to ensure credibility and enforcement of the emission data.

### Trusted consortium hub

The TCH manages processed data from the ESU and integrates it with a smart contract embedded in the Polygon blockchain network. The PUC Endorsement algorithm, executed every 15 min, automates emission validation for Petrol/CNG and Diesel vehicles, forwarding compliant data to the Predictive Analytics Engine while flagging non-compliant vehicles for authorities, restricting their fuel and insurance access. Users initiate certification monthly, with data analyzed in under 15 min. The MVIs at RTOs validate vehicle conditions and digitally sign endorsements using the El Gamal algorithm, ensuring secure storage within the blockchain. The edge node, equipped with GPS, captures the vehicle owner’s precise location, allowing each transaction to be categorized into a specific regional transaction pool. Within this framework, every region is managed by a designated MVI who acts as the sole validator for that region. This localized validation mechanism enables the Regional MVI to promptly verify transactions and generate blocks without the need for multi-node consensus. As a result, block creation becomes significantly faster, ensuring efficient and region-specific processing of emission endorsements. Compliant users earn redeemable tokens for fuel, insurance, and maintenance services, while non-compliant vehicles are restricted from accessing these services, encouraging participation. To ensure endorsement validity, the PUC Verification Algorithm is used, operating in two stages: first, it decrypts and checks emissions data against predefined limits using the El Gamal algorithm; second, it verifies the MVI’s digital signature. Invalid endorsements are removed, and flagged vehicles undergo further review, maintaining a secure and transparent emission monitoring system.

**Fig. 3:**
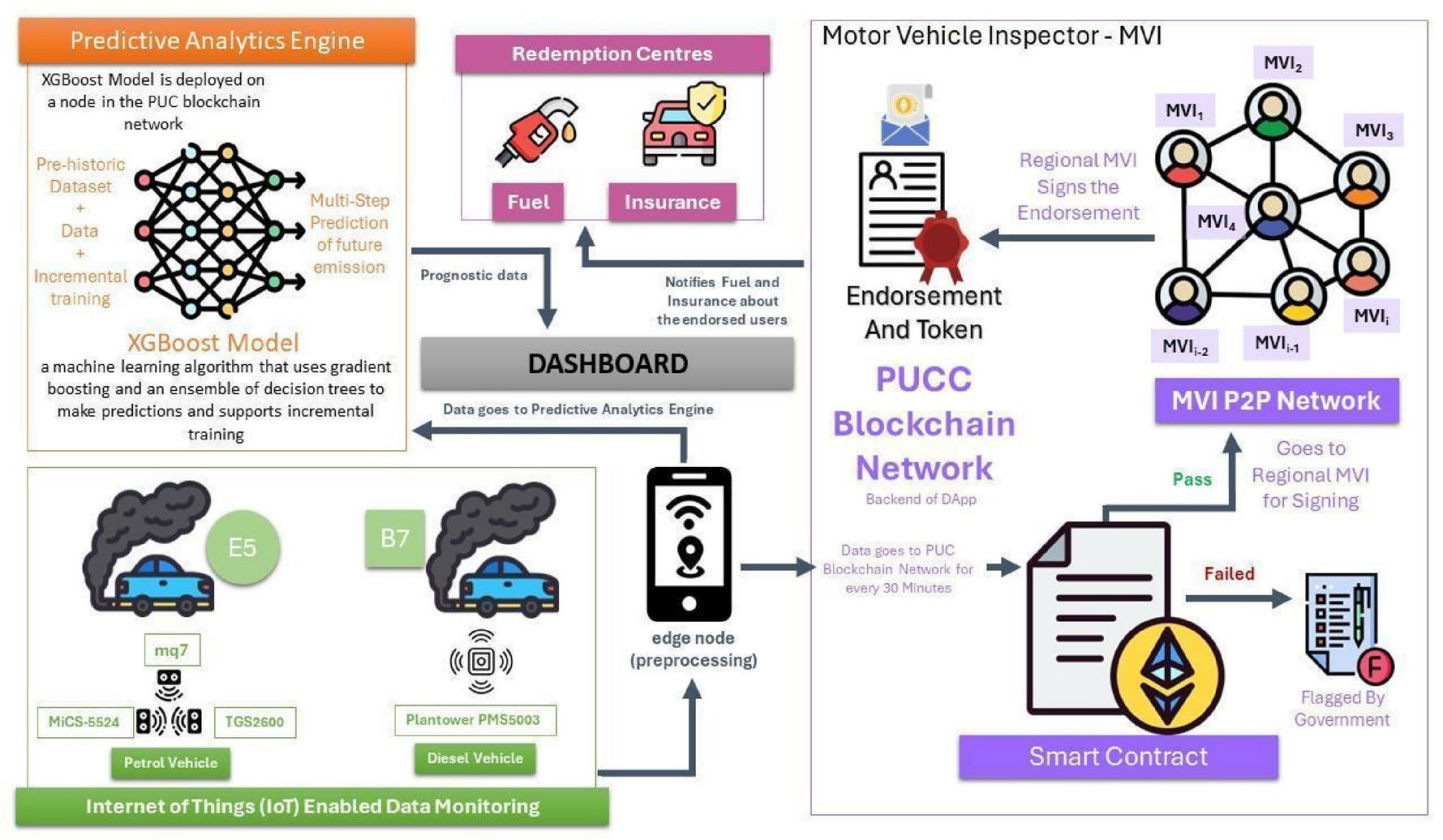
Blockchain-based vehicle emission monitoring-system side view.

In a real-world scenario, a diesel vehicle owner initiates emission testing at the start of the month. The ESU detects particulate matter using the Plantower PMS5003 sensor, transmitting processed data to the edge node, which filters and forwards it to the TCH. The PUC Endorsement algorithm validates the data, and if compliant, the Regional MVI digitally signs the endorsement, storing it securely on the blockchain. Compliant owners receive redeemable tokens, while non-compliant vehicles are flagged, restricting access to fuel and insurance services. The Polygon network ensures high scalability, low costs, and energy efficiency, while the El Gamal algorithm secures digital signatures. The modular design allows seamless expansion to accommodate new vehicle types, IoT sensors, and regional MVIs, ensuring a secure, scalable, and tamper-proof emission monitoring system.

The Polygon offers significant scalability, low gas fees, and faster transaction speeds, making it an ideal solution for high-throughput applications. Its seamless compatibility with Ethereum allows developers to easily deploy decentralized applications (dApps) while benefiting from enhanced transaction speeds and reduced costs. Polygon also supports a customizable consensus mechanism, allowing developers to tailor it to specific needs for optimized performance. So, it is useful to deploy our customized consensus algorithm and enhance the energy efficiency of the network, ensuring a more environmentally friendly solution compared to traditional Proof-of-Work systems and other blockchains. The system, as illustrated in Fig. 3, closely represents the overall flow of operations from the system’s perspective by integrating multiple components to create an end-to-end, decentralized emissions monitoring and compliance framework. It begins with IoT sensors mounted on vehicles that continuously collect real-time emission data such as CO, NOx, and CO₂ levels. This data is transmitted to an edge device, typically a smartphone, which performs preprocessing tasks including cleaning, filtering, and aggregation to ensure only relevant and high-quality data is retained. Once processed, the emission data is securely stored on a blockchain using smart contracts, which automatically execute key actions like validation and reward distribution. The predictive analytics engine, powered by the XGBoost algorithm, analyzes historical emission patterns to forecast future pollution levels and provides timely maintenance recommendations to the vehicle owner. Meanwhile, MVIs participate in a P2P network where they validate digital endorsements and contribute to consensus, ensuring data reliability without central authority. The smart contracts manage rule enforcement, schedule maintenance notifications, and handle reward mechanisms. As a compliance incentive, vehicle owners who maintain eco-friendly performance are granted redeemable benefits such as discounts on fuel or vehicle insurance through designated Redemption Centers, making the system not only technically efficient but also practically rewarding for users.

The TCH combines smart contracts, localized validation, and token incentives on Polygon to ensure secure, scalable, and accountable emission data endorsement. The next section introduces the Predictive Analytics Engine for trend forecasting.

### Predictive analytics engine

Lack of awareness about emissions and timely maintenance poses challenges in pollution control. To address this, the framework integrates a Predictive Analytics Engine using the XGBoost model, known for its high accuracy in multi-step time series predictions. This model forecasts emission patterns and optimal maintenance intervals, ensuring proactive intervention. Trained on a historic dataset from the Vehicle Certification Agency (UK) via Kaggle, the model is fine-tuned for precise emission predictions. It also supports incremental training, appending new blockchain-verified data to improve real-time adaptability, making the system more effective and dynamic in monitoring vehicle emissions. The model processes cleaned and validated sensor data at the edge node and feeds its predictions into the compliance decision logic, which is then stored immutably on the blockchain. This seamless flow ensures each component contributes meaningfully to accurate forecasting and traceable decision-making within the system.

#### Dataset description

The dataset includes information about 6,756 vehicles, featuring key attributes essential for analyzing vehicle emissions.

**Fig. 4:**
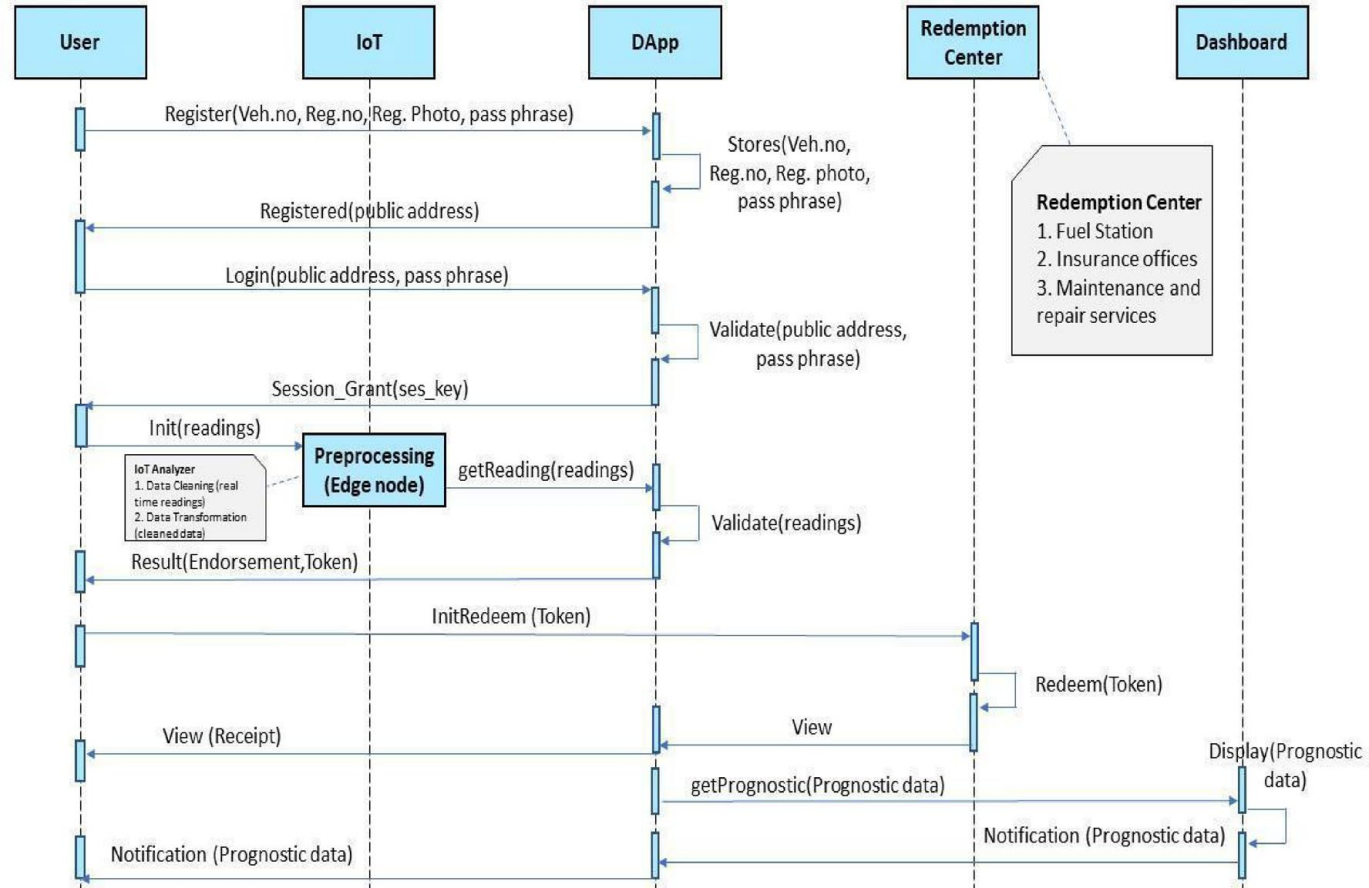
Client-side sequence flow.

Each vehicle is identified by a unique car_id and details such as manufacturer, model, and description. It specifies the transmission type, which includes the transmission_type categorized as “Manual,” “Automatic,” or “Electric - Not Applicable.” Additional fields encompass engine_size_cm3, indicating engine displacement, fuel type (e.g., Petrol, Diesel), powertrain system, power_ps measured in metric horsepower, and co2_emissions_gPERkm, representing CO2 emissions. The preprocessing steps involved addressing missing values in the transmission_type field where the electric vehicles were filled with “Automatic” due to their lack of traditional transmission systems. Similarly, null values in engine_size_cm3 were replaced with “0.0” for electric vehicles, reflecting their absence of combustion engines. The null values in power_ps were filled with “0.0” where CO2 emissions were also “0.0,” in line with the characteristics of electric vehicles that typically produce no direct emissions. The inconsistencies in transmission_type and fuel_type for a subset of petrol-powered vehicles were rectified through manual validation to enhance data integrity. The outliers with low CO_2_ emissions associated with high-power vehicles were identified and corrected by cross-referencing external data sources^28^. The XGBoost model was trained using 80% of the dataset, while the remaining 20% was reserved for testing to evaluate prediction accuracy. Grid search was employed for hyperparameter tuning, optimizing key parameters such as learning rate, max depth, and the number of estimators. To enhance model robustness and prevent overfitting, 5-fold cross-validation was applied during training. This approach enabled the model to effectively learn emission patterns and forecast potential violations within the proposed framework. Once established, the XGBoost model processes real-time data from the blockchain network every 15 min, applying filtering and limit checks before incorporating it into predictions. Using parameters like fuel type, model type, and engine type, it compares current readings with historic data to forecast future emission patterns. The forecasted data is then analyzed by a decision tree model on the edge node to predict the next maintenance interval. If a critical issue is detected, users receive instant notifications via dashboard updates and SMS, ensuring timely maintenance. This system enhances user awareness, promotes proactive vehicle care, and helps maintain environmental compliance.

**Fig. 5:**
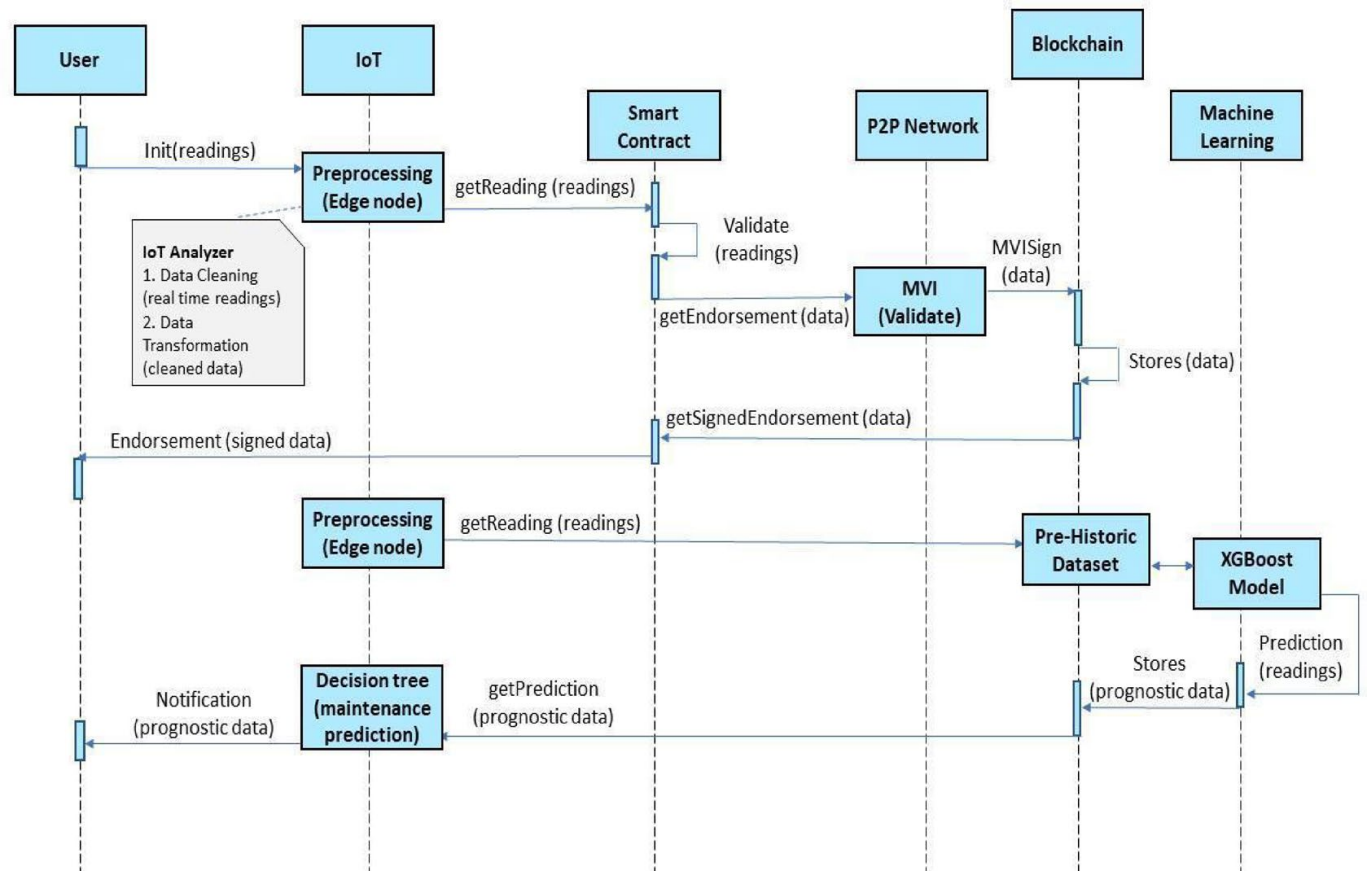
System-side sequence flow.

To ensure accurate forecasts, the predictive analytics engine incorporates anomaly detection to handle edge cases like incomplete data or sudden emission fluctuations. When anomalies are detected, the system requests additional data from the user or flags it for MVI review, ensuring prediction reliability. These validation mechanisms enhance the accuracy, robustness, and trustworthiness of the framework in real-world scenarios. Figures 4 and 5 illustrate the sequence diagrams of the DApp framework from both client-side and system-side perspectives. The process starts with user registration, where vehicle data is stored on the blockchain. IoT devices collect emission readings, which are preprocessed by an edge node before validation by the DApp. Once validated, the system generates an endorsement and token, which users can redeem for services like fuel discounts or maintenance. Simultaneously, an MVI endorses the data, and the XGBoost model analyzes it to provide predictive maintenance recommendations, notifying users via the dashboard.

In summary, the Predictive Analytics Engine uses an XGBoost model trained on historic and real-time blockchain-verified data to accurately forecast vehicle emission trends and maintenance needs. This enables proactive interventions and adaptation via anomaly detection and incremental learning. The subsequent section presents the experimental evaluation of the proposed framework, demonstrating its effectiveness in real-world scenarios.

## Analytical model description

In the proposed system, two smart contracts are deployed at the Ethereum Virtual Machine (EVM) node within the blockchain network to automate and streamline emission monitoring and management. These smart contracts are designed to ensure the seamless functioning of the system to support the core algorithms, each addressing a specific functionality of the framework: a) ESU Installation, b). PUCE algorithm, c). PUC verification algorithm (PUCV).

Algorithm 3 outlines the procedure for installing the ESU, a key component in the proposed emission monitoring framework that enables real-time pollutant detection and secure data integration. The algorithm begins by retrieving vehicle-specific information—fuel type, engine type, and model—from the OBU. Based on the fuel type, the system selects and installs an appropriate set of gas sensors tailored for monitoring relevant emissions. These sensors are integrated into the ESU, which is securely connected to the vehicle. To ensure data integrity and traceability, a cryptographic hash is generated using SHA-256 algorithm from the vehicle details and sensor configuration, and recorded on the blockchain. The ESU is then activated with a predefined sampling schedule to begin continuous data collection. This process ensures that the sensing mechanism is vehicle-specific, tamper-proof, and seamlessly integrated into the system’s edge and blockchain infrastructure. The algorithm contributes to the overall framework by enabling automated, secure, and scalable emission tracking, addressing major limitations of traditional Pollution Under Control (PUC) systems and supporting more effective environmental regulation.

**Algorithm 3:**
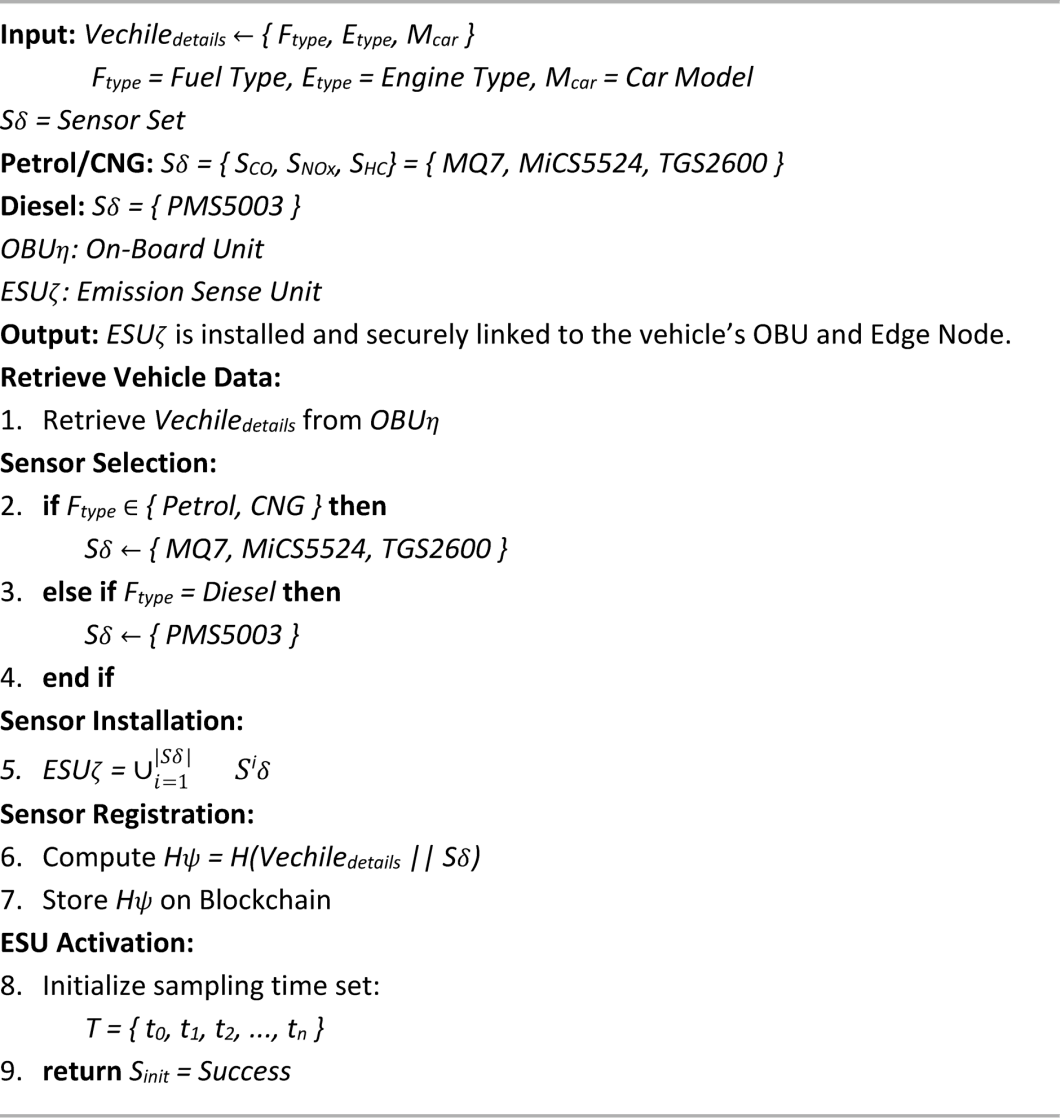
Emission sense unit (ESU) installation.

The selection of appropriate sensors is Important for reliable emission monitoring in our blockchain-IoT framework. Our sensor selection methodology follows a systematic multi-criteria decision analysis approach, evaluating cost-effectiveness, technical specifications, availability, power efficiency, and integration complexity. This evidence-based selection ensures optimal performance-per-dollar while meeting emission detection requirements for large-scale deployment.

### Sensor selection rationale

The selection of MQ-7, MiCS-5524, TGS2600, and PMS5003 sensors was based on multi-criteria optimization prioritizing cost-effectiveness, power efficiency, and scalability for developing economies. The chosen sensors consume < 100 mA collectively, enabling integration with smartphones or Arduino microcontrollers without external power supplies, unlike alternatives such as OPC-N3, which require dedicated power sources^37^. The cost analysis reveals significant advantages of 10-20x cost reduction, enabling large-scale deployment in developing regions where affordability is critical for widespread adoption. The field validation studies in Beijing traffic conditions and Bengaluru urban environments demonstrate the practical reliability of these sensors in real-world vehicle emission monitoring scenarios as presented in Table 6. Our selected sensor suite achieves 71.4% cost reduction compared to premium alternatives while maintaining good to very good accuracy levels. This cost optimization enables large-scale deployment without compromising measurement quality, enhancing the accessibility of vehicle emission monitoring through blockchain-IoT integration.

**Table 6:** Summary of sensor selection rationale and alternatives.

Sensor	Target pollutant(s)	Selection rationale	Specifications	Alternatives	Pros & cons of alternatives
MQ-7	Carbon monoxide (CO)	High sensitivity; low cost; suitable temp & power	10 − 1,000 ppm range; 30 s response	SGX Electrochemical, Bosch CO	SGX: higher accuracy, expensive; Bosch: limited supply
MiCS5524	Multi-gas (CO, ethanol, H2, NH3, CH4)	Multi-gas detection; compact; mid-cost; low power	CO: 1–1,000 ppm; size 5 × 7 × 1.55 mm	SGX variants, sensirion SGP30	SGX: costly; SGP30: digital but costlier; multiple sensors increase complexity
TGS2600	Hydrocarbons (HC)	Sensitive; low power; small size	1–30 ppm range; fast response	TGS2602, SGX HC, Bosch BME680	TGS2602: better VOC selectivity, costlier; BME680: complex, limited stock
PMS5003	Particulate matter (PM1, PM2.5, PM10)	Validated accuracy; fast response; cost effective	Particle size 0.3–10 μm; <10s response time	Sensirion SPS30, OPC-N3, SDS018	SPS30: more precise, costly; OPC-N3: expensive; SDS018: variable performance

The selected sensors comply with emission monitoring standards with MQ-7 detects CO with ± 5 ppm accuracy aligned with World Health Organization (WHO) guidelines, while MiCS5524 effectively monitors main automotive gases. Together, they ensure reliable detection within regulatory thresholds for vehicle emissions.

Algorithm 4 details the PUCE, which verifies a vehicle’s compliance with emission standards and facilitates secure certification within the proposed blockchain-based framework. This multi-step process integrates data acquisition, validation, and endorsement mechanisms to ensure trustworthy pollution monitoring. The algorithm begins with data collection and processing (4.1), where emission readings from gas sensors installed via the ESU are captured, preprocessed at the edge node, encrypted using AES-256, and forwarded to the TCH. A cryptographic hash with the SHA-256 algorithm of the encrypted data is also stored on the blockchain for immutability and auditability. Next, the PUC Endorsement Execution module (4.2) decrypts the emission data and compares it with predefined pollutant limits. Based on compliance status, a smart contract is triggered to either validate the vehicle or flag it for violations. The compliance result, linked with vehicle details, is then securely recorded on the blockchain. Finally, the endorsement and digital signing step (4.3) involves manual authorization by a local MVI, who reviews the emission data and digitally signs the certification using ElGamal encryption. This signed certificate is permanently stored on the blockchain, ensuring authenticity and traceability. By combining automated sensing, secure data handling, regulatory oversight, and decentralized storage, the PUCE algorithm enhances the credibility, efficiency, and transparency of emission certification, overcoming the major weaknesses in conventional PUCC systems.

**Algorithm 4:**
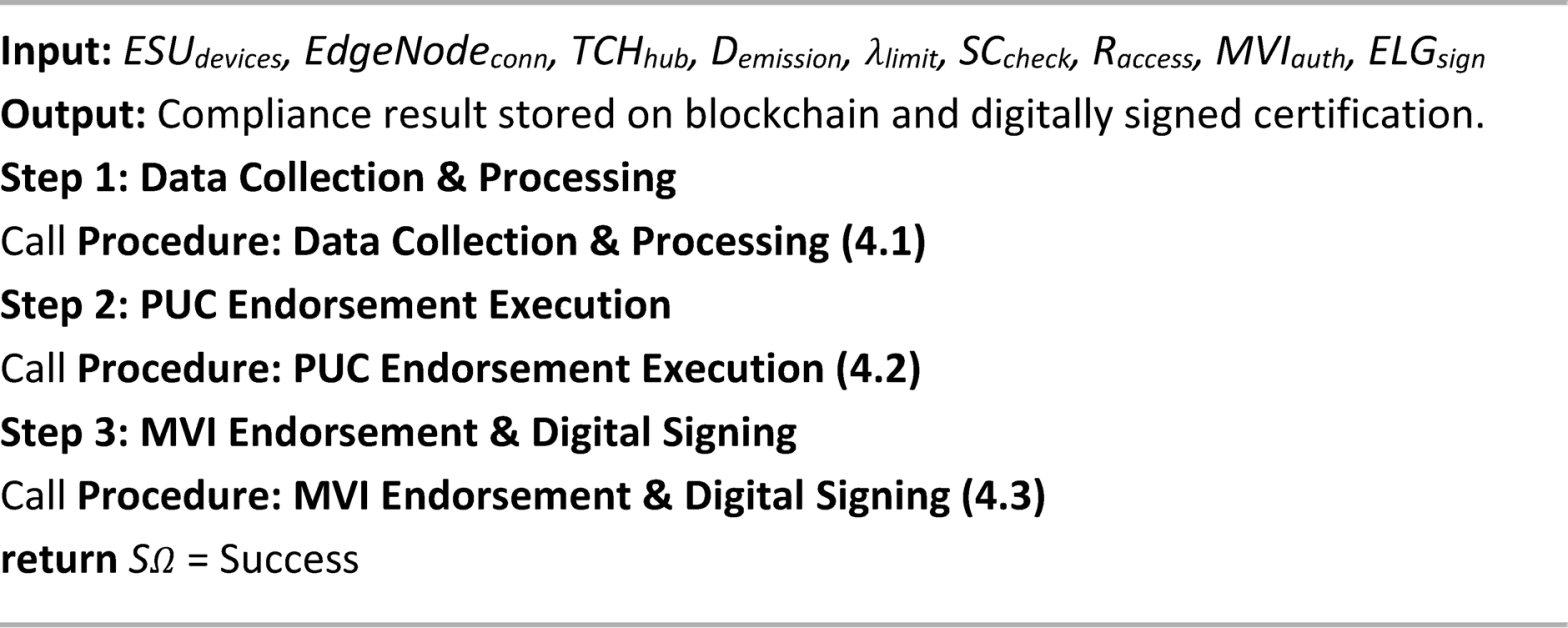
PUC endorsement algorithm (PUCE).

**Algorithm 4.1:**
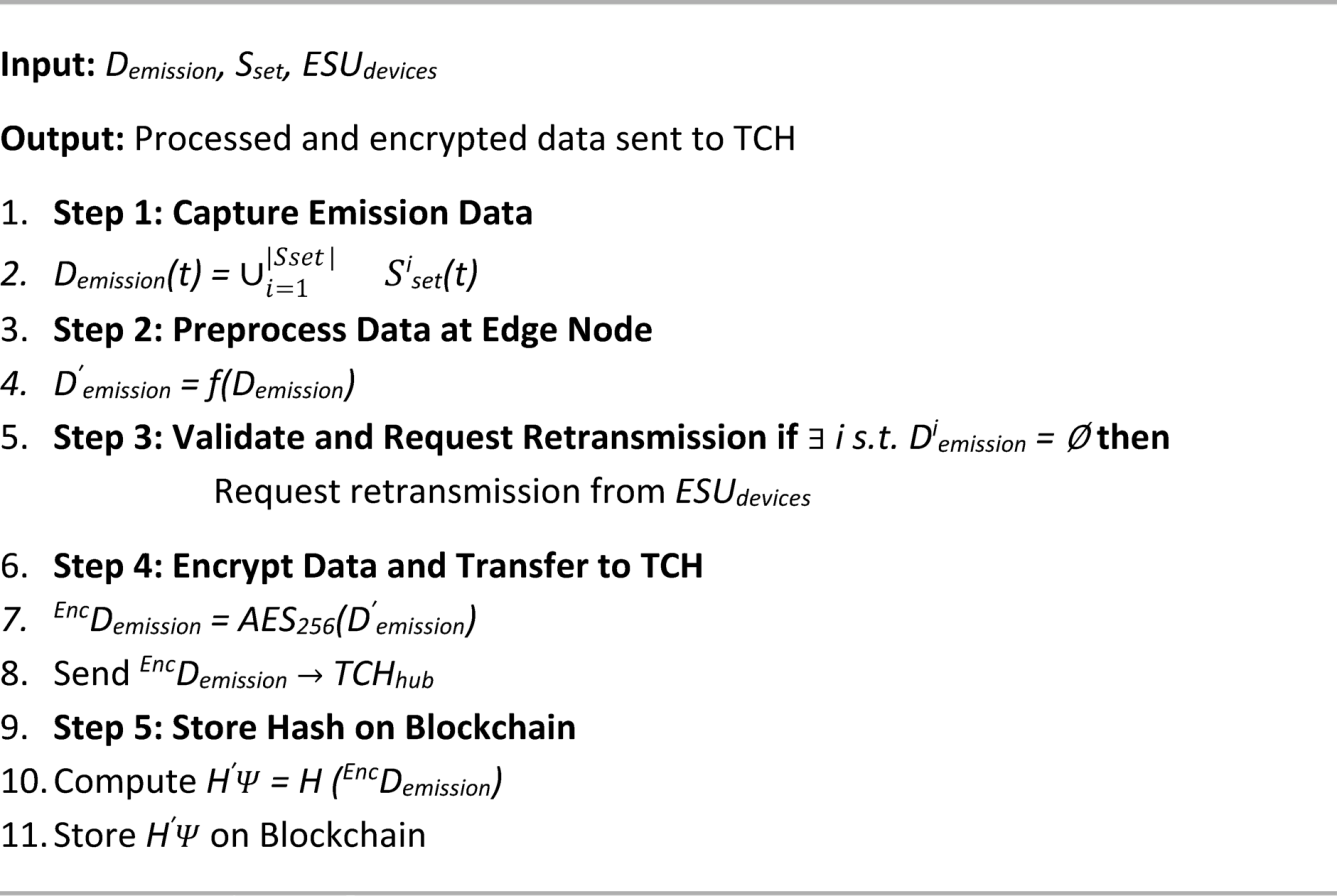
Data collection & processing.

**Algorithm 4.2:**
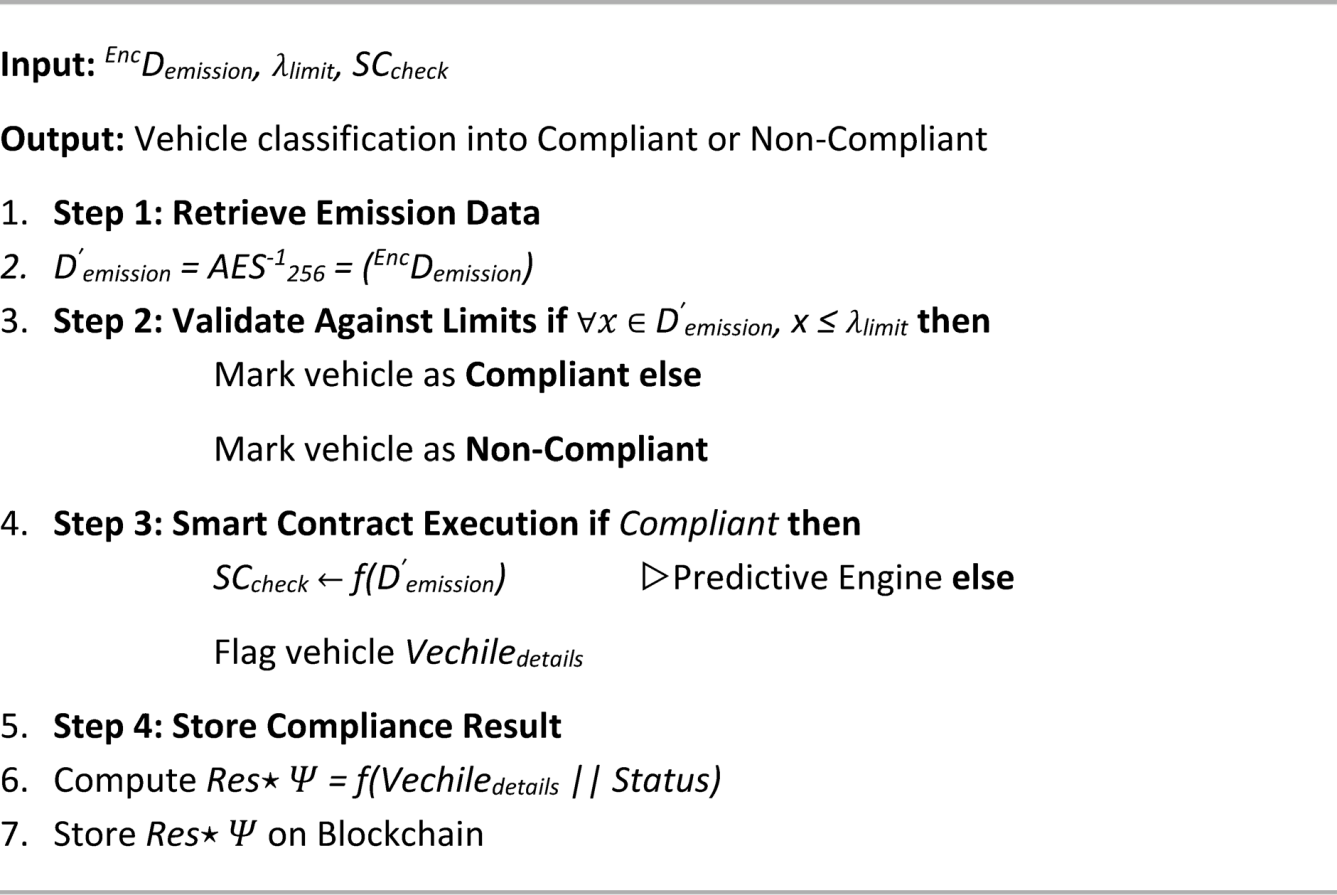
PUC endorsement execution.

**Algorithm 4.3:**
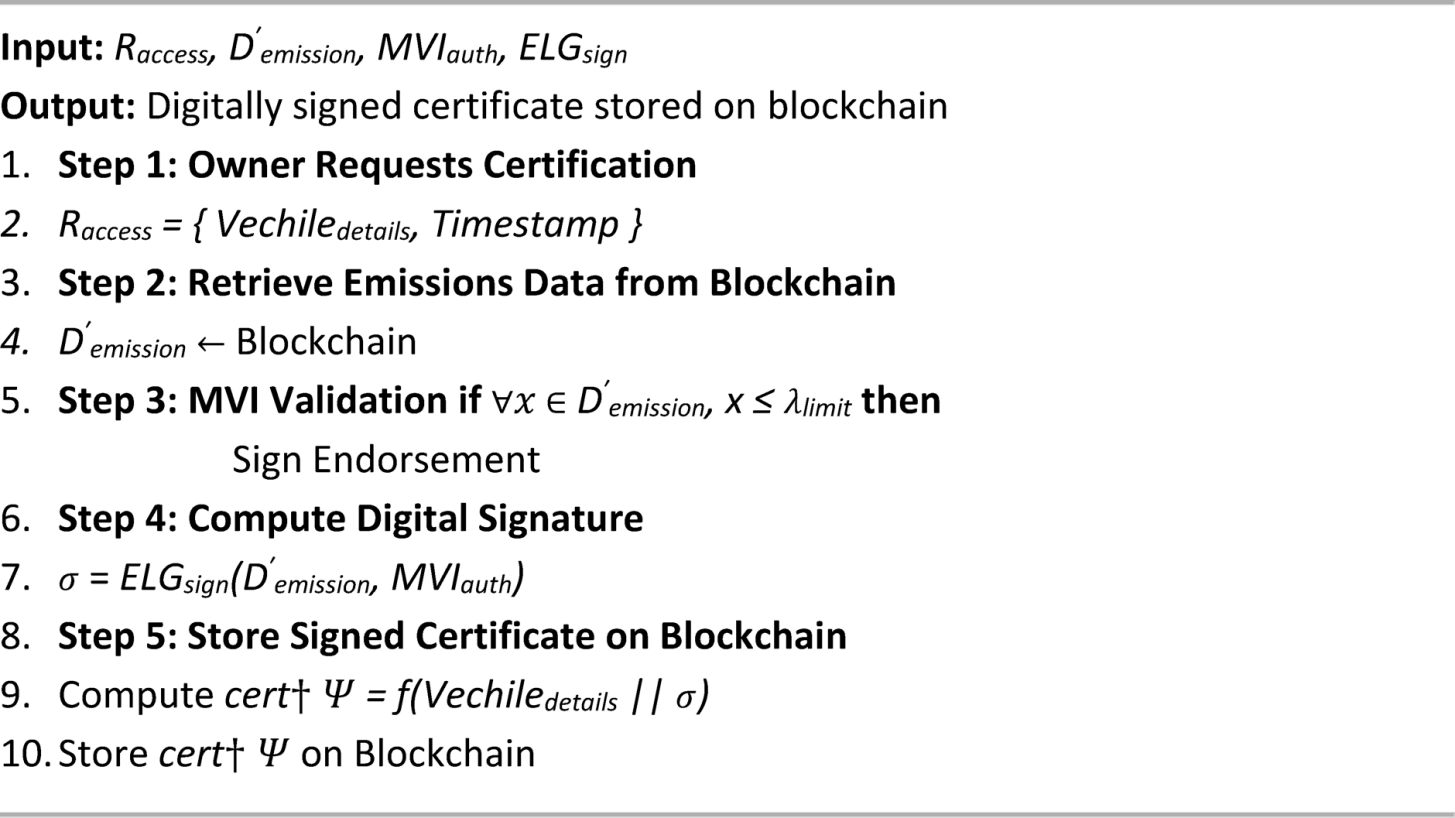
MVI endorsement & digital signing.

Algorithm 5 outlines the PUCV algorithm, which ensures the authenticity of a digitally signed Pollution Under Control (PUC) certificate within the decentralized framework. The process begins with the retrieval of the digital signature embedded in the certificate and decryption of the emission data using the public ElGamal key. The decrypted data is then validated against predefined emission standards to determine compliance. If all parameters fall within acceptable limits, the vehicle is marked as valid; otherwise, it is flagged as non-compliant. To ensure data integrity, the signature of the MVI is verified by computing a cryptographic hash that links the emission data with the MVI authorization credentials. Finally, the verification result is stored immutably on the blockchain. Compliant vehicles retain their endorsements, while non-compliant entries are removed and flagged for regulatory action. This algorithm plays a critical role in maintaining trust, security, and regulatory compliance across the emission monitoring ecosystem by validating both data integrity and endorsement authenticity.

**Algorithm 5:**
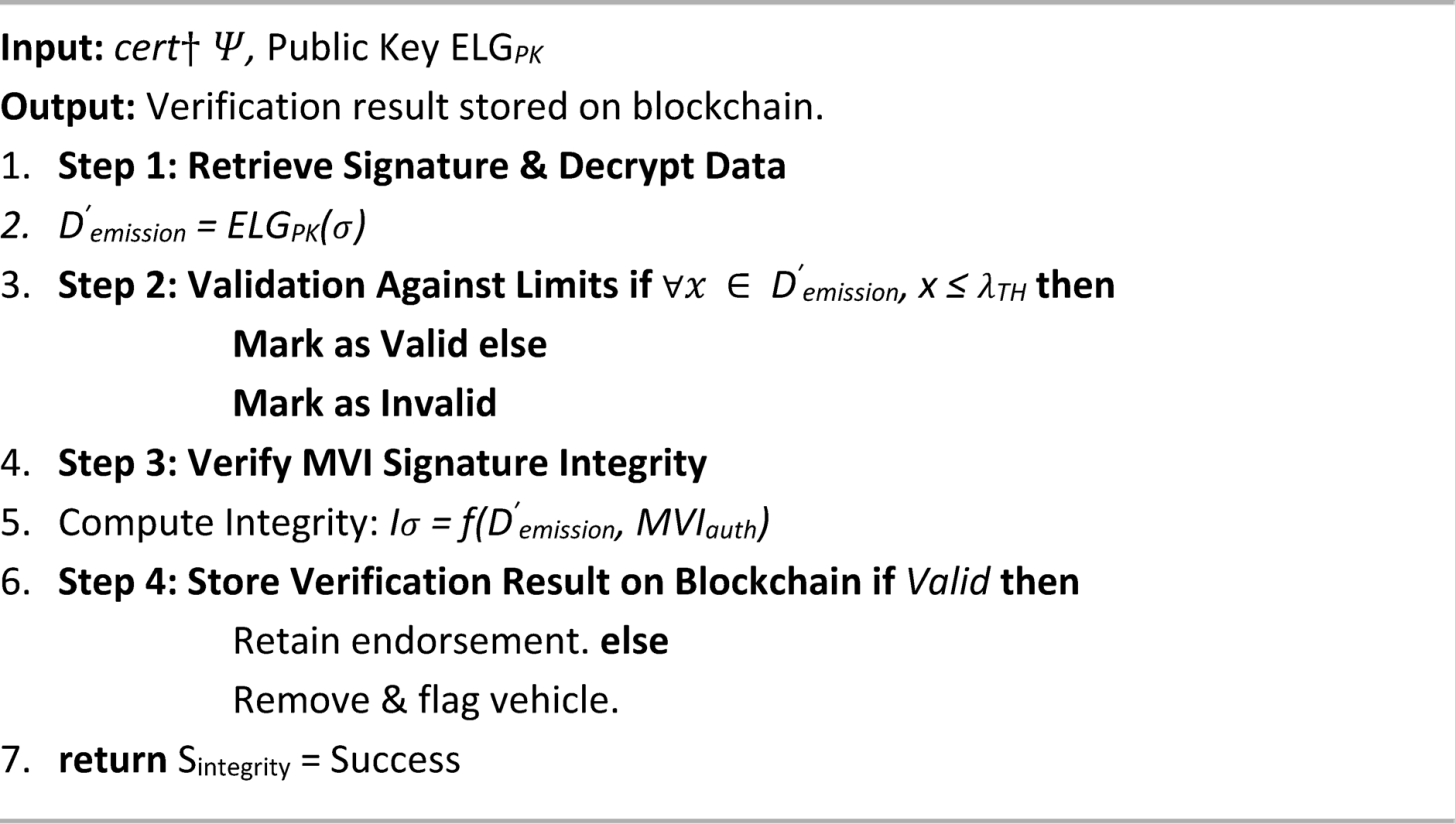
PUC verification algorithm (PUCV).

The system deploys two smart contracts on the EVM to automate vehicle emission monitoring through three algorithms: ESU Installation (sensor setup and hash logging), PUC Endorsement (data validation, certification, and on-chain storage), and PUC Verification (signature authentication, compliance checks, and violation flagging), ensuring secure, tamper-proof, and scalable regulatory enforcement.

## Applied implementation

The proposed vehicle emission monitoring framework follows a modular, multi-layered approach encompassing system setup, sensor-based data acquisition, blockchain-enabled compliance verification, and predictive analytics. The system begins with the registration of each vehicle, where unique identifiers, such as vehicle number and location, are hashed and registered on a blockchain network using MetaMask and elliptic curve cryptography. The SHA-256 hash function is used to generate unique hash values for each emission data record that forms a tamper-proof digital fingerprint, ensuring data integrity and immutability once stored on the consortium blockchain. This approach ensures that any future retrieval or validation of emission data can be efficiently verified against its blockchain-stored hash, preventing unauthorized modifications. Based on fuel type, emission gases like CO, NOx, HC, and PMx are tracked using appropriate gas sensors connected via Arduino UNO and integrated into an IoT board. These are linked to a smartphone that forwards emissions data over Wi-Fi 6 to the blockchain and analytics layers. The Ethereum-based DApp, developed with Solidity and ReactJS, enables secure smart contract execution for automatic PUC issuance and verification. The emission data is encrypted, hashed, and stored on-chain, ensuring tamper-proof compliance records. Smart contracts classify vehicles as compliant or non-compliant, and Motor Vehicle Inspectors (MVIs) can digitally sign verified certificates using cryptographic keys stored securely.

The framework uses only those data records whose hashes have been validated on the blockchain, ensuring that the input to the XGBoost-based Predictive Analytics Engine is authentic and unaltered. This integration of blockchain-verified data into the analytics pipeline enhances the trustworthiness of emission trend predictions and supports proactive maintenance recommendations. The prototype uses accessible, cost-effective components and software tools, including open-source simulators, low-cost IoT devices, and free blockchain infrastructure. While the current implementation relies on resources available within our resource limits, the framework is flexible and can be adapted or expanded. Although our present study is simulation-based, the framework is purpose-built for real-world deployment, with important considerations addressed:

### Scalability

Since the system is a distributed, modular architecture comprising edge IoT nodes and a partitioned blockchain supports horizontal scalability, enabling the management of thousands of vehicles by distributing workload across administrative regions. The simulation results confirm the technical feasibility of securely connecting thousands of vehicles.

### Latency and real-time performance

To mitigate blockchain-induced latency, the framework utilizes a hybrid data strategy: emission data is pre-processed at the edge, and only summarized, verified records are stored on-chain. This approach, supported by lightweight consortium blockchains and edge/fog computing, achieves near real-time processing with response times under 100 ms in our tests, which is critical for high-traffic urban environments. The sub-chain handling of raw sensor streams further reduces bottlenecks, while on-chain data remains tamper-evident and auditable.

### Legal and regulatory factors

The deployment at scale requires strict compliance with data privacy laws, cross-border data sharing regulations, and environmental standards. We are actively engaging with government agencies and smart city stakeholders to co-develop regulatory frameworks and pilot the system in operational suburban settings, facilitating regulatory alignment and stakeholder adoption.

### Implementation roadmap

Recognizing that these challenges are non-trivial, our solution adopts a phased rollout strategy. Initial pilot studies with public sector partners will serve as testbeds for validating performance, optimizing scalability, and refining compliance mechanisms. This incremental approach supports progressive integration into existing urban infrastructure and regulatory ecosystems, ensuring both technical robustness and policy alignment.

## Experimental evaluation and setup

### Simulation environment

The simulation environment for vehicle emissions monitoring uses SUMO, configured to replicate real-world traffic dynamics and emission profiles. The HBEFA model simulates pollutants like CO2, CO, NOx, HC, and PMx for Petrol, Diesel, and CNG vehicles, with emissions tracked at every 0.25-second time step over 15 min. To improve data granularity, the time step was reduced to 0.1 s, and the PHEM model was integrated with HBEFA for a broader range of emission factors. This dual-model approach ensures accurate emissions tracking, aligning with IoT-based data collection intervals for synchronized real-time monitoring.

**Fig. 6:**
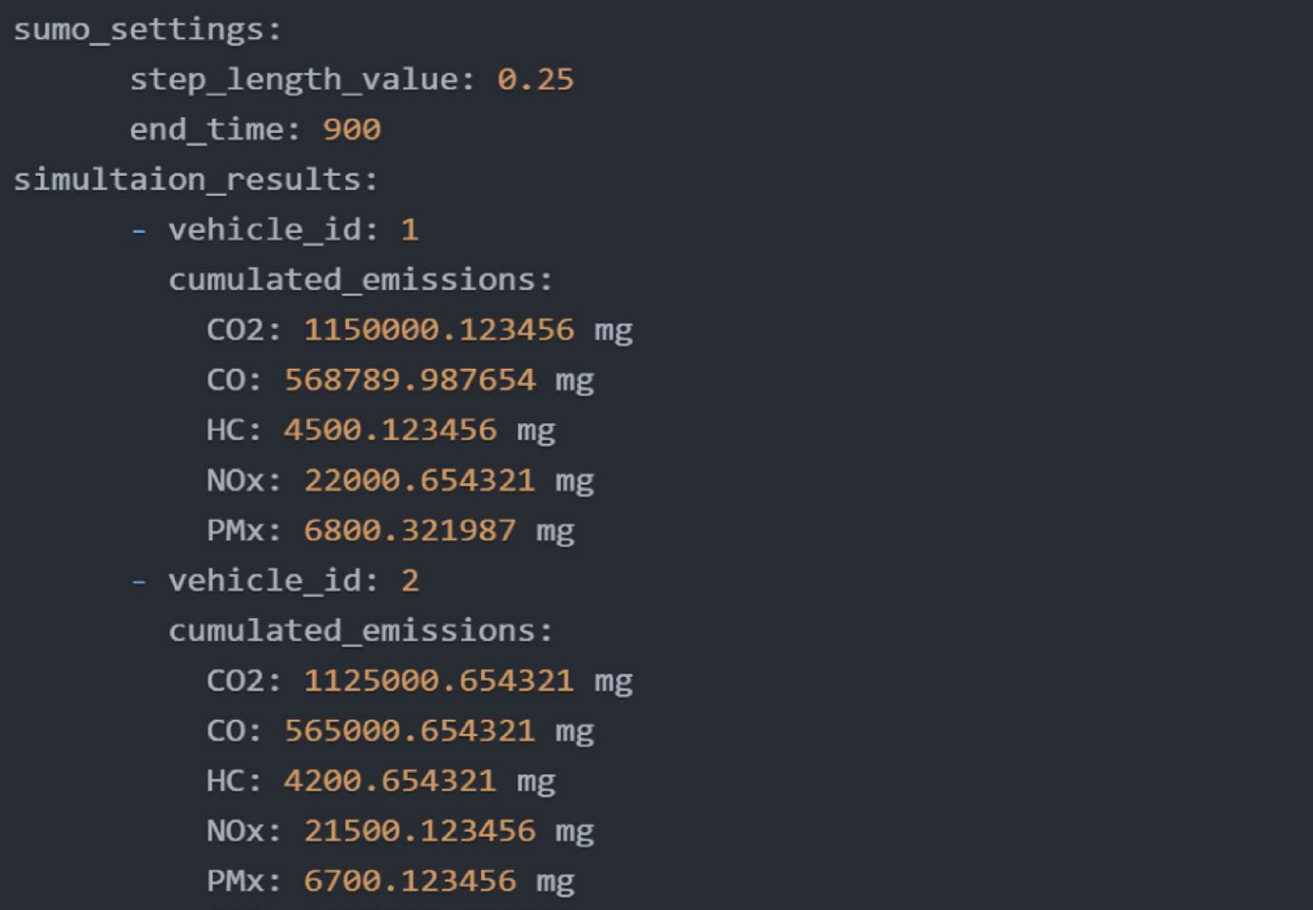
Simulation results in SUMO.

Due to limited prior work for direct comparison, Fog Computing and Cloud Computing environments were simulated as baselines to evaluate the proposed system’s performance, latency, energy consumption, and network efficiency. In the Fog Computing simulation, NS3 models a network of fog nodes that preprocess IoT emissions data before forwarding relevant information to the cloud, reducing latency and bandwidth usage. Key parameters include processing delay, energy consumption, and data aggregation efficiency, with communication simulated using 802.11ax Wi-Fi and LTE networks. In the Cloud Computing simulation, NS3 emulates a centralized cloud system processing large-scale IoT data, utilizing high-bandwidth TCP/IP networks. The simulation assesses latency, task processing time, energy consumption, and data throughput, incorporating a dynamic task offloading mechanism to optimize cloud scalability. These simulations provide insights into performance trade-offs and optimization strategies for integrating real-time emissions monitoring with IoT and edge computing technologies.

### Emission monitoring prototype

The IoT system utilizes the Photon IoT device for seamless connectivity and user-friendly operation, with built-in Wi-Fi for easy setup and cost efficiency. The user’s smartphone acts as the edge node, running on Android with a Snapdragon 900 MHz processor and 1 GB RAM, supporting Wi-Fi 6 (802.11ax) for efficient data transmission. The system integrates MQ-7 (CO), MiCS-5524 (NOx), TGS2600 (HC for Petrol/CNG), and Plantower PMS5003 (Diesel) sensors, connected via an Arduino UNO R3 to manage data flow. An LCD provides real-time CO and NOx readings for instant emission monitoring as shown in Table 7.

**Fig. 7:**
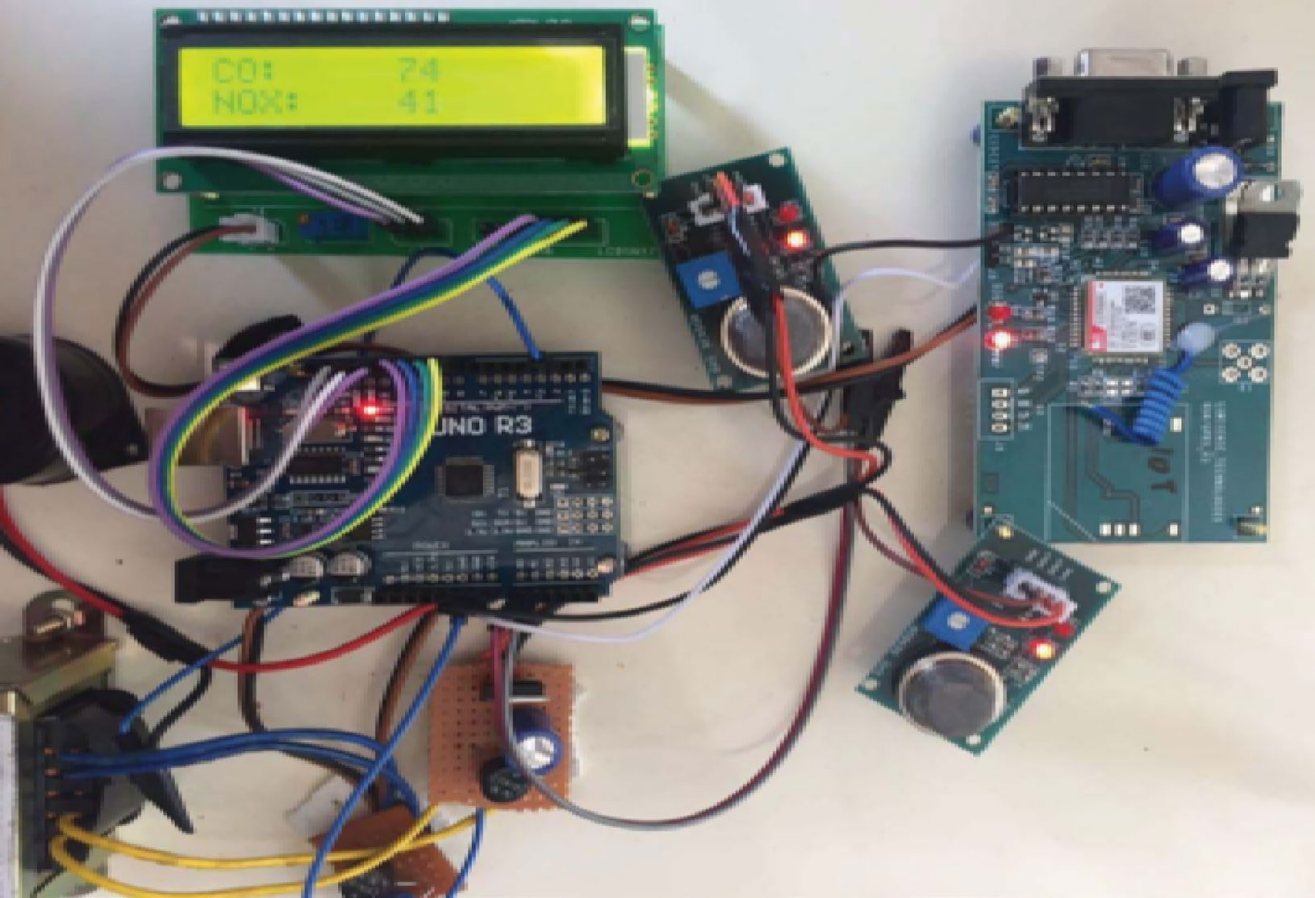
Prototype setup for sensor data processing.

**Fig. 8:**
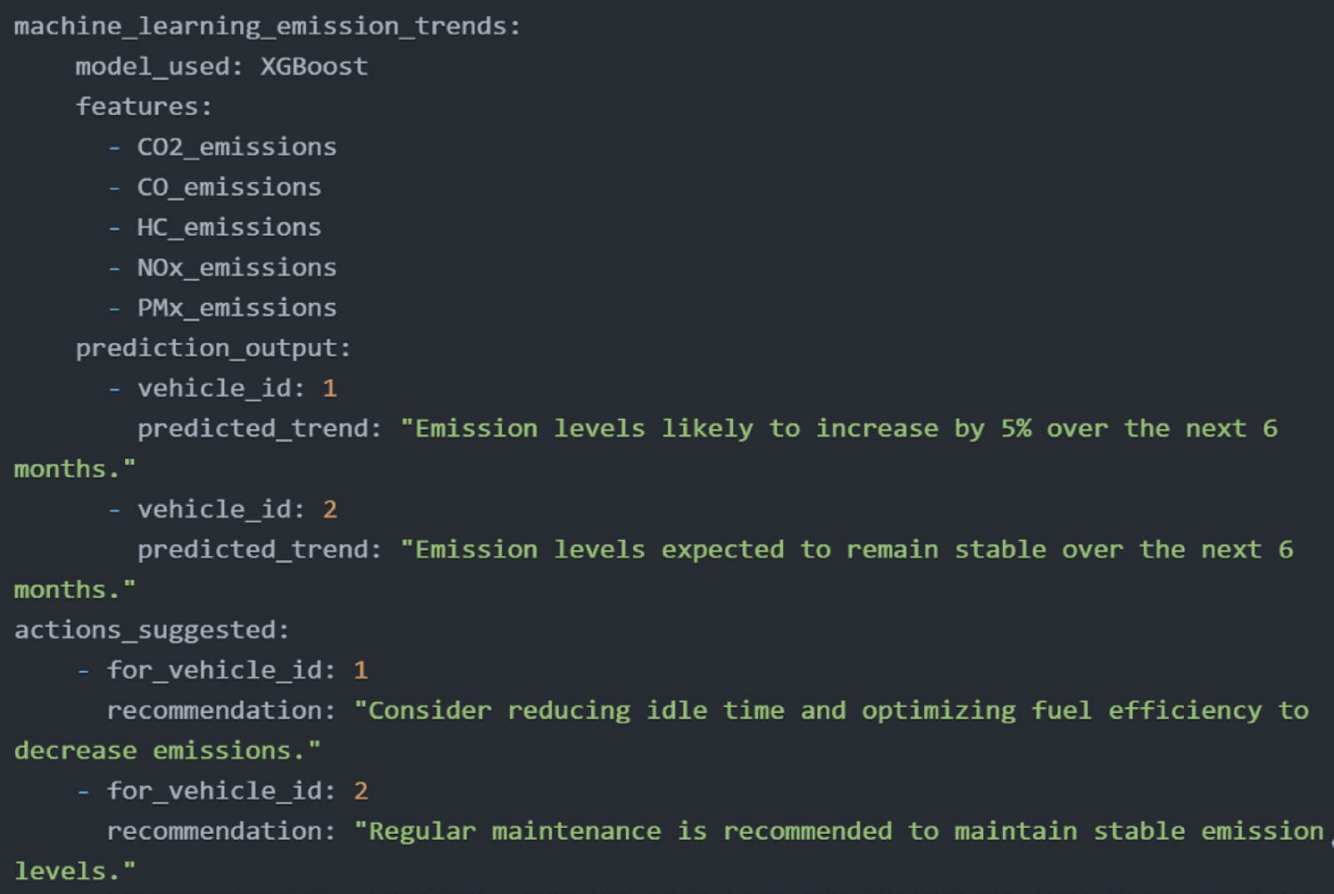
Predictive analytical engine.

The simulation results from SUMO presented in Fig. 6 illustrates the cumulative emissions for two different vehicles - Vehicle A (a 2015 model petrol hatchback) and Vehicle B (a 2012 model diesel sedan) - monitored over 900 s with a 0.25-second step length, measuring CO₂, CO, HC, NOₓ, and PMx. Vehicle 1 and Vehicle 2 were selected to simulate real-world variations in vehicle conditions rather than type alone. While both vehicles belong to similar categories in terms of size and usage, Vehicle 1 exhibited higher emissions of CO₂, CO, and PMx, primarily due to inadequate maintenance and infrequent servicing. In contrast, Vehicle 2, maintained according to recommended schedules, emitted slightly higher HC and NOₓ levels, which can be attributed to normal combustion behavior. This comparison underscores how maintenance quality significantly affects emission levels, reinforcing the framework’s ability to detect and differentiate real-time emission discrepancies for targeted mitigation. Figure 7 presents a prototype monitoring system using an Arduino microcontroller, various sensors, an LCD for real-time readings, and a GSM module for wireless data transmission and remote monitoring. Figure 8 displays XGBoost model predictions, forecasting a 5% CO2 increase for Vehicle 1 over six months, suggesting idle time reduction and fuel efficiency optimization. Vehicle 2’s emissions remain stable, with recommendations for regular maintenance to sustain low emissions, aiding fleet managers in emission reduction and fuel efficiency improvements.

**Table 7:** Development environment.

Components	Devices	Specifications
Hardware	Photon IoT	Processor: ARM Cortex-M3 120 MHz
		Memory: 1 MB flash, 128 KB RAM
	Smartphone	Android device
		Processor: snapdragon 900 MHz
		Memory: 1 GB
Connectivity standard	Wi-Fi module	Wi-Fi 6 (802.11ax)
Library and framework	Python API libraries	
Resources	MQ-7 MiCS-5524 TGS2600 **Petrol/CNG**	
	Plantower PMS5003 **Diesel**	

The DApp framework is built on Ethereum, utilizing Solidity for secure and efficient smart contracts that operate autonomously. These contracts, containing core business logic, are deployed using JavaScript for seamless interaction with the client interface. To enhance scalability and reduce costs, the Polygon network is integrated as a layer-2 solution without compromising security. The client-side framework is developed in React JS, ensuring a dynamic and responsive user interface for an improved user experience. This combination of Ethereum, Polygon, Solidity, JavaScript, and React JS creates a robust, efficient, and scalable platform for the DApp, as shown in Table 8.

The emission monitoring system was simulated using SUMO with HBEFA and PHEM models to capture detailed real-world pollutant emissions at fine time intervals. Fog and Cloud computing environments were simulated with NS3 to benchmark latency, energy use, and network efficiency, highlighting the benefits of edge processing for real-time data. The prototype used Photon IoT devices and sensors (MQ-7, MiCS-5524, TGS2600 for Petrol/CNG, PMS5003 for Diesel) connected via Arduino and a smartphone acting as an edge node with Wi-Fi 6. Real-time emissions from two vehicles demonstrated how maintenance impacts pollutant levels, with the XGBoost model forecasting future emissions and maintenance needs. The DApp runs on Ethereum with Solidity smart contracts and uses the Polygon network for scalable, low-cost transactions, paired with a React JS client interface for user interaction. This setup ensures a robust, scalable, and secure platform for vehicle emission monitoring and management.

**Table 8:** Blockchain development environment.

Components	Specifications
Processor configuration	Pre-selected validators: Intel Core i7, 4 cores @ 1.30 GHz
	Peers: Intel Core i5, 2 cores @ 3.4 MHz
Memory configuration	Pre-selected validators: 32 GB RAM
	Peers: 8 GB RAM
Operating systems	Windows 11, 64-bit
DApp framework	Polygon ethereum network
Languages	Solidity, javascript
Library support	React JS

## Results and discussion

The results and discussion section analyzes the performance of cloud computing, fog computing, and blockchain environments, evaluating efficiency and scalability. Simulations in NS3 for fog and cloud computing, along with Polygon blockchain for the blockchain setup, are conducted.

A comparison of accuracy of XGBoost and F-measure across cloud and blockchain platforms highlights its reliability in processing IoT data. Key metrics such as throughput, response time, and processing time are examined, along with the correlation between validator count and confirmation time in blockchain, providing insights into system performance across different computational setups. The accuracy measures the correctly predicted outcomes. On comparing the accuracy of the XGBoost across blockchain and cloud platforms is crucial to assess reliability. While blockchain offers decentralization, cloud platforms provide high computational power, impacting model performance. This analysis explores how these environments influence predictive accuracy, highlighting their strengths and limitations for IoT data processing.

**Fig. 9:**
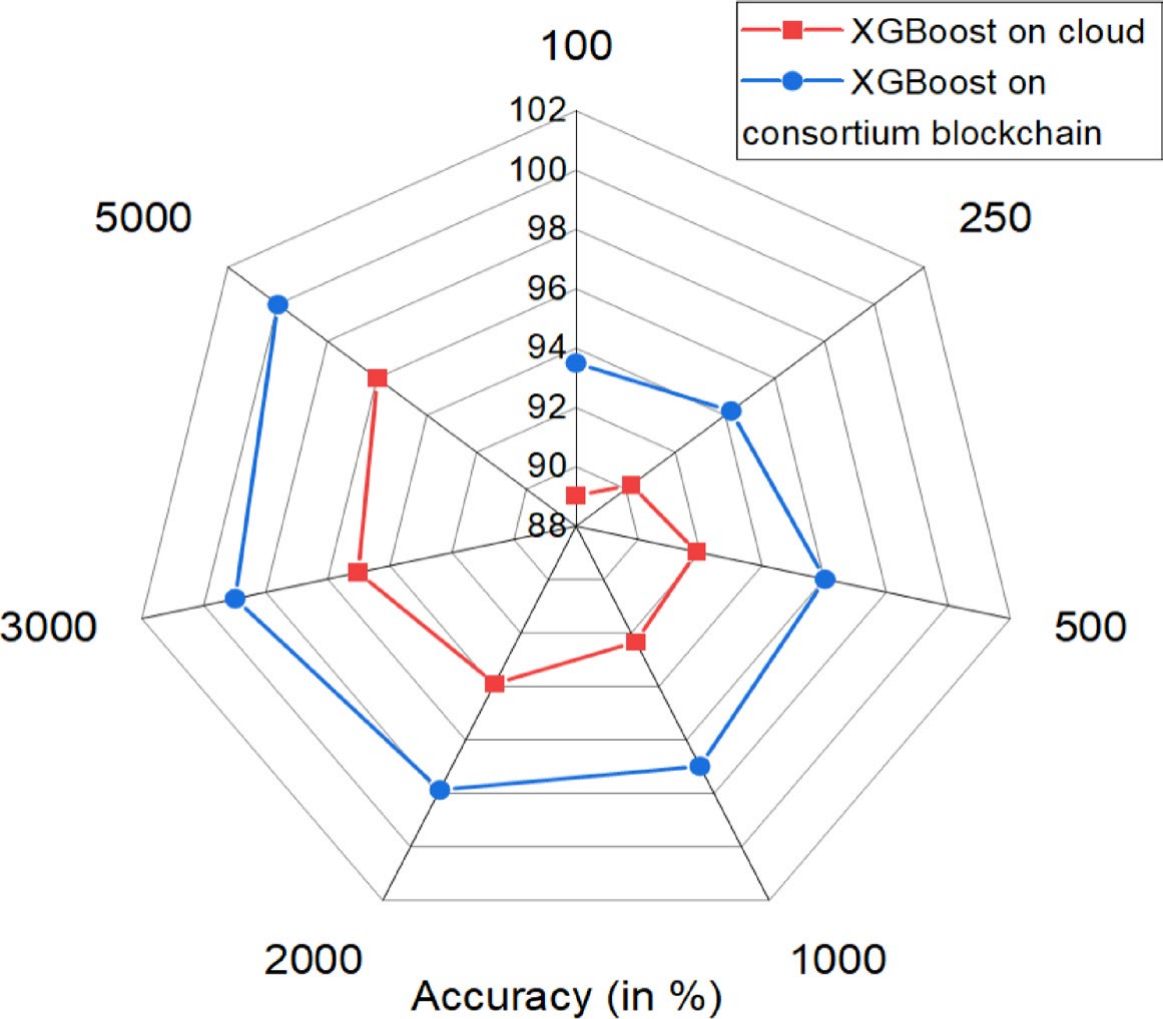
XGBoost on blockchain network vs. the cloud platform based on the accuracy.

Figure 9 compares XGBoost accuracy on a cloud platform and a consortium blockchain with edge transfer. The blockchain model consistently outperforms the cloud, with a 4.47% higher accuracy for 100 records and 4.006% higher for 5000 records, demonstrating better scalability. The decentralized framework enhances accuracy through collaborative training, leveraging contributions from consortium members to improve predictive performance. The proposed blockchain-based solution overperformed existing paradigms across multiple dimensions. It achieves up to 11% higher prediction accuracy, a 90% reduction in response time, and 4–7 times higher throughput compared to traditional PUCC systems with relational databases. Additionally, it ensures tamper-proof data integrity and full audit transparency, effectively eliminating fraud and manipulation risks as summarized in Table 9.

We compared the accuracy of two different systems—blockchain-based and cloud-based—by evaluating their performance in processing IoT data. The assessment considered key performance metrics, including true positives, false positives, true negatives, and false negatives, across multiple datasets. By analyzing how each system handled data classification, we identified variations in predictive reliability. The blockchain-based system demonstrated higher accuracy due to its decentralized validation process, which enhances data integrity and reduces errors. In contrast, the cloud-based system exhibited slight fluctuations in accuracy due to centralized processing constraints. This comparison highlights the strengths and limitations of each system, providing insights into their suitability for real-world applications.

**Table 9:** Comparison of proposed blockchain framework with fog, cloud, and traditional PUCC systems.

Metric	Blockchain framework (proposed)	Fog computing	Cloud computing	Traditional database (pucc)	Improvement over traditional pucc
Prediction accuracy (%)	97.98–99.99	-	95.52	88–92	11% higher
F-measure (%)	99.41	-	97.36	89–91	10% higher
Throughput (Mbps) (at 5000 recs)	384.8–679	~ 290	~ 200	< 100	4-7x higher
Response time (ms)	62.8–91.98.8.98	68.4	669.1	> 1000	90% faster
Processing time growth (% increase)	219.6–225.9.6.9	1737.7	1718.1	> 2000	85% lower
Communication cost (bits)	2900	over 4780	over 4780	over 6000	50% lower
Data integrity	Tamper-proof, immutable	Moderate (edge risk)	Moderate (cloud risk)	Prone to tampering	Fully tamper-proof
Transparency	Full audit trail	Partial	Partial	Limited, opaque	Full, real-time transparency
Fraud/manipulation	Nearly impossible	Possible at edge	Possible at central node	Manual override	Eliminates fraud risk

**Fig. 10:**
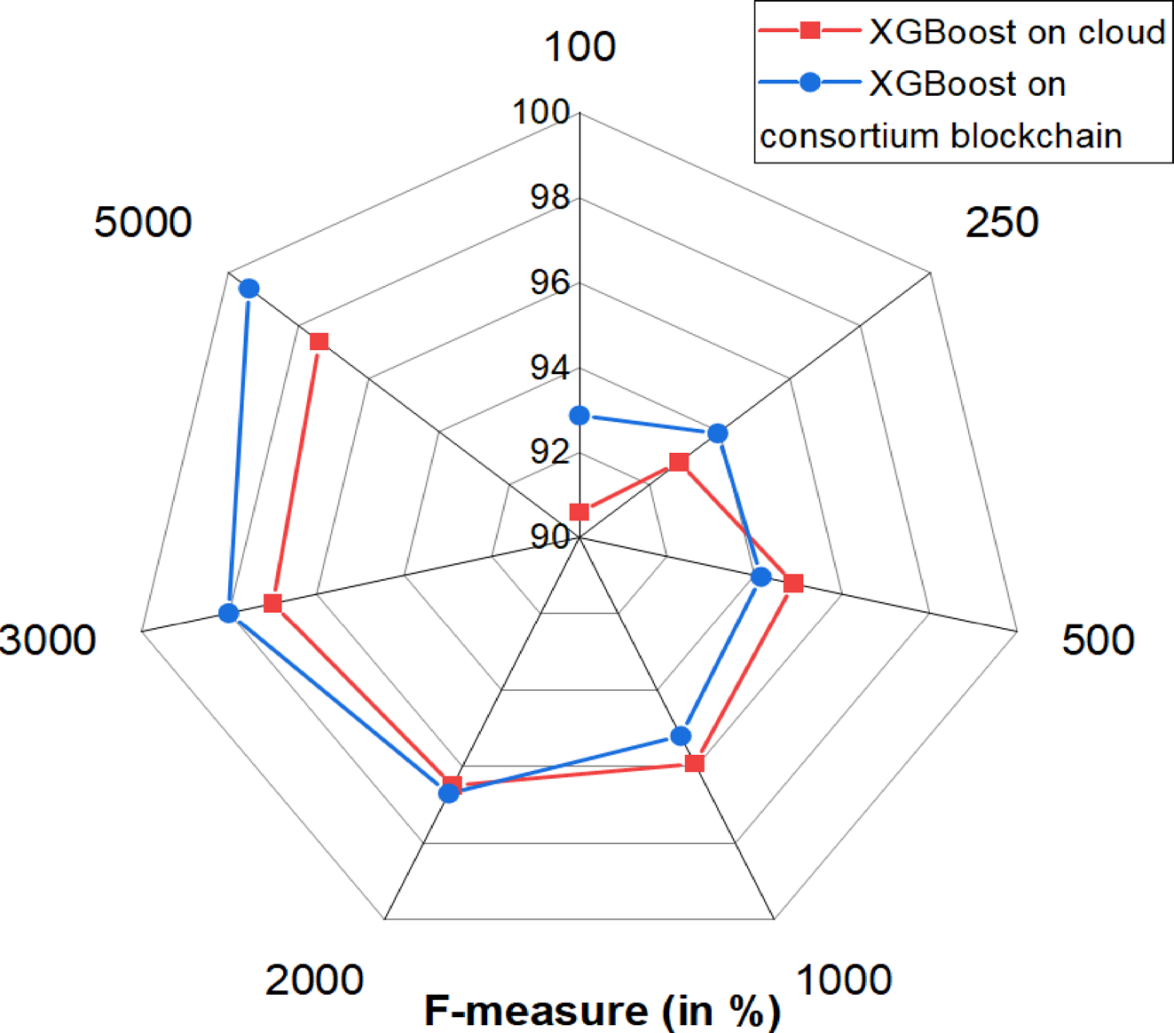
XGBoost on blockchain network vs. cloud platform based on the F-measure.

The F-measure, the harmonic mean of precision and recall, evaluates the balance between false positives and false negatives, particularly in imbalanced datasets. Comparing the F-measure of XGBoost on blockchain and cloud platforms helps assess their impact on prediction reliability, highlighting the strengths and limitations of decentralized and centralized infrastructures in handling IoT data and machine learning tasks. Figure 10 presents a comparative analysis of the F-measure performance of XGBoost on a blockchain network and a cloud platform across various record counts. At 100 records, the blockchain model outperforms the cloud by 2.4746%, maintaining an advantage of 1.1770% at 250 records. As the record count increases to 500, the difference reduces to 0.7777%, yet the blockchain model continues to perform better by approximately 0.78%. At 1000 records, the trend remains consistent, with a slight edge for the blockchain model. By 2000 records, the difference diminishes further, but at 3000 records, the performance advantage rises again to 1.0389%. The most significant difference occurs at 5000 records, where the blockchain model surpasses the cloud by 2.0528%. These results indicate that while the performance gap varies with dataset size, XGBoost on the consortium blockchain consistently demonstrates superior F-measure performance, particularly with larger datasets, highlighting its potential for improved efficiency in handling extensive data.

The F-measure of blockchain-based and cloud-based system by evaluating their precision and recall in processing IoT data. Precision measures the proportion of correctly predicted positive cases, while recall indicates the ability to identify all actual positive cases. By analyzing these metrics, we assessed the trade-off between accuracy and completeness in both systems.

**Fig. 11:**
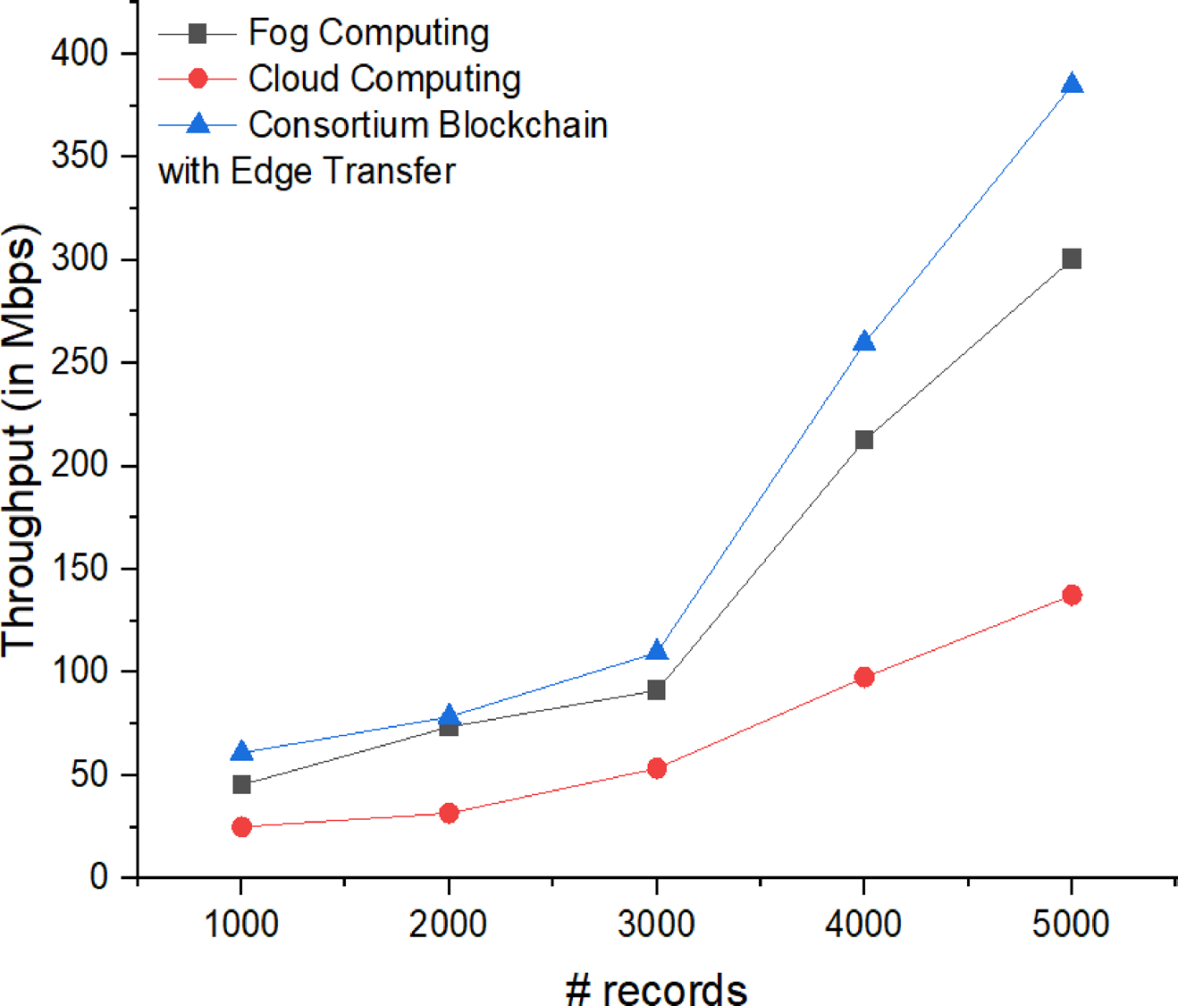
Throughput for fog computing, cloud computing, and blockchain.

The blockchain-based system demonstrated a higher F-measure due to its decentralized validation, ensuring more reliable data classification. In contrast, the cloud-based system exhibited slight variations, influenced by centralized processing and potential latency. This comparison highlights the strengths of each system in handling IoT data, providing their effectiveness for real-world applications. Throughput measures the amount of data successfully processed or transmitted within a system over time, reflecting its efficiency and capacity. Comparing throughput across fog computing, cloud computing, and blockchain systems is crucial to assess their scalability, responsiveness, and suitability for real-time applications. This study analyzes throughput to determine how each system handles IoT-driven data, identifying the optimal framework for high-speed, large-scale data processing in dynamic environments.

The correlation between fog, cloud computing, and consortium blockchain networks with edge nodes was analyzed, revealing a positive correlation between throughput and record volume, indicating effective workload handling. Studies^22,23^ show that fog computing consistently achieves the highest throughput across varying record counts. However, Fig. 11 highlights that consortium blockchain surpasses fog computing in scalability, with throughput increasing from 61.08 Mbps (1000 records) to 384.824 Mbps (5000 records). While fog computing excels in high-throughput localized processing and cloud computing offers flexibility, consortium blockchain balances security, decentralization, and scalability, making it highly suitable for our applications.

The throughput of two blockchain-based, fog-based, and cloud-based systems was compared by analyzing their data processing efficiency and transmission speed, considering factors such as processing delays and network latency. The blockchain-based system exhibited lower throughput due to the overhead of consensus mechanisms, which introduce additional delays. In contrast, the cloud-based system demonstrated higher throughput, benefiting from centralized processing and optimized resource allocation. This comparison highlights the trade-offs between decentralization and speed, offering insights into the suitability of each system for handling real-time IoT data.

The response time measures the duration between a request and its response, impacting real-time applications like IoT. The lower response times are important for latency-sensitive tasks such as vehicle emissions monitoring.

**Fig. 12:**
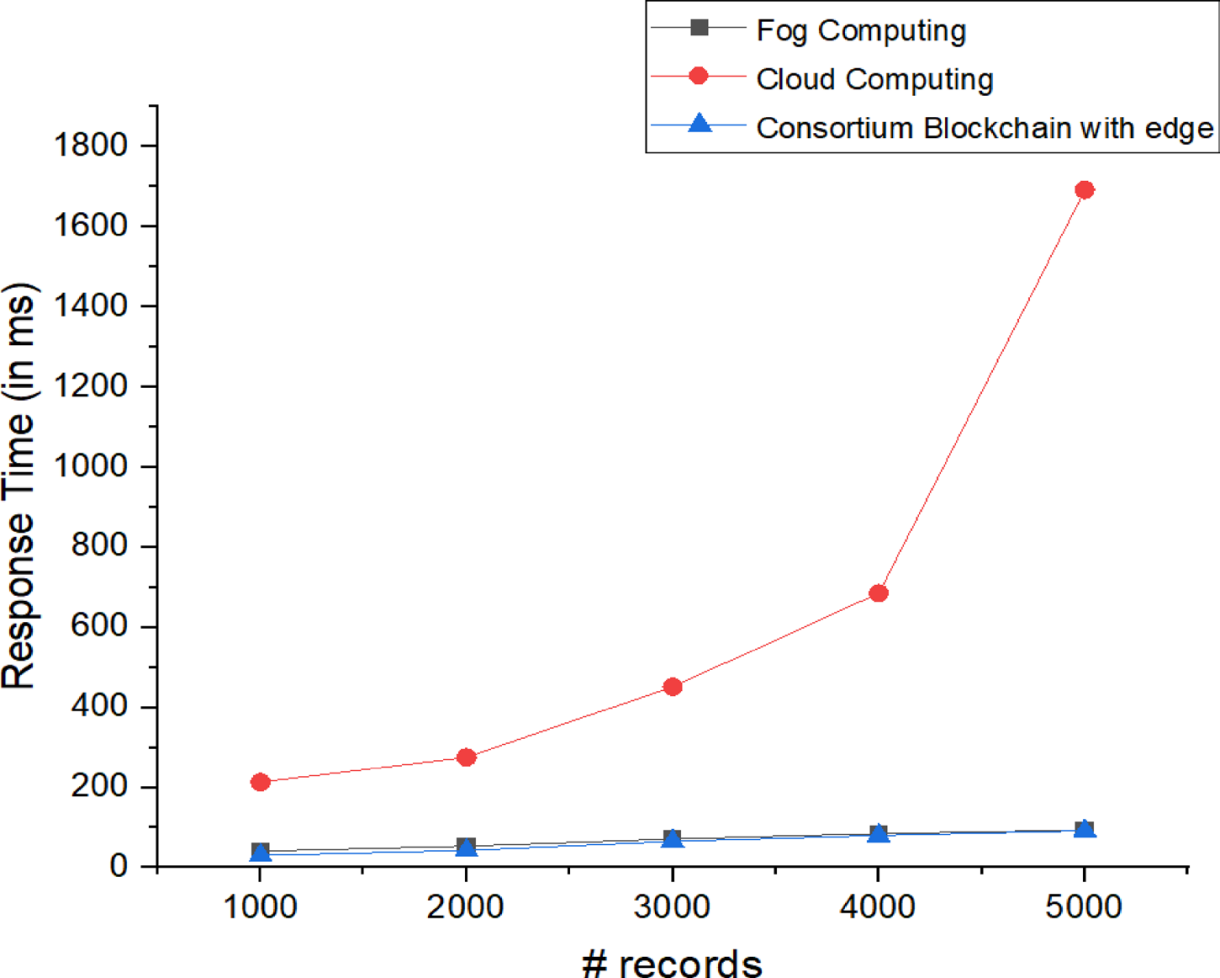
Response time for fog computing, cloud computing, and blockchain.

In this study, NS3 simulations were used to analyze response times across fog computing, cloud computing, and blockchain. The studies^22,23^ indicate that fog computing has the shortest response time, while cloud access is the longest as depicted in Fig. 12. However, our framework shows that consortium blockchain with an edge device achieves comparable response times to fog computing. The mean response times observed were 62.8196 ms for blockchain, 68.354 ms for fog, and 669.06 ms for cloud computing, with standard deviations of 22.874, 21.764, and 623.98, respectively, highlighting the efficiency of consortium blockchain in latency-sensitive environments.

Table 10 Presents network latency, the time taken for data to travel from the IoT network to the consortium blockchain. IoT devices (DEV1, DEV2, DEV3) transmit sensor data to the edge node with latencies of 20 Ms, 22 Ms, and 21 Ms, respectively. The edge node then forwards data to blockchain validators (VAL-ID1, VAL-ID2) with latencies of 51 Ms and 55 Ms. Additionally, communication between VAL-ID1 and VAL-ID2 incurs a latency of 10 Ms, highlighting the overall transmission delays in the system.

**Table 10:** Network testbed configuration.

Source	Destination	Latency (in ms)
IoT device (DEV1)	Edge node	20
IoT device (DEV2)	Edge node	22
IoT device (DEV3)	Edge node	21
Edge node	Pre-selected validator (VAL-ID1)	51
Edge node	Pre-selected validator (VAL-ID2)	55
Pre-selected validator (VAL-ID1)	Pre-selected validator (VAL-ID2)	10

**Fig. 13:**
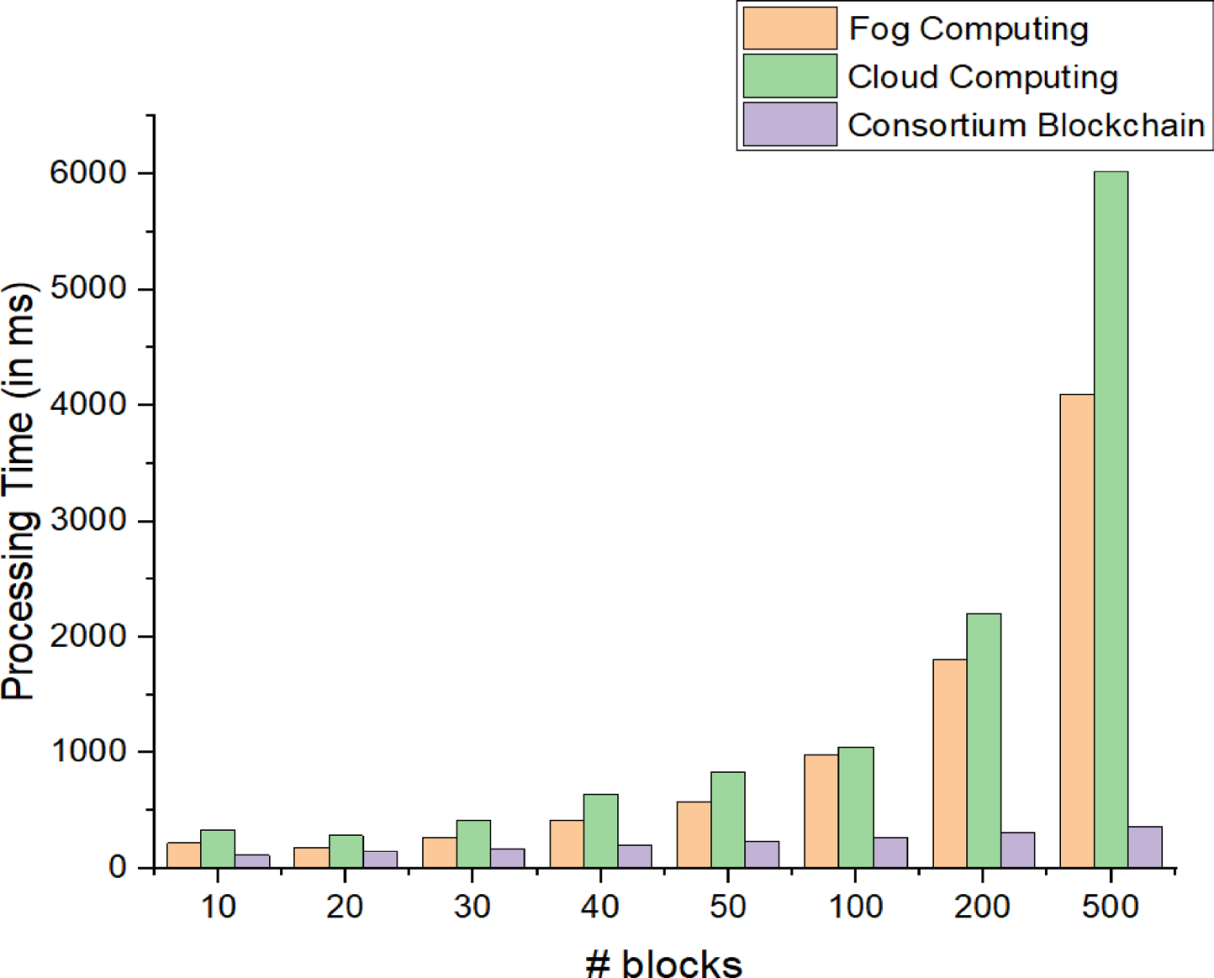
Processing time for fog computing, cloud computing, and blockchain.

The processing time measures the duration required to complete computational tasks, impacting the efficiency of fog, cloud, and blockchain systems, especially in real-time IoT applications. Lower processing time signifies faster execution and better performance. In this study, NS3 simulations were used to measure processing times under varying workloads and network conditions. Comparing these times highlights each system’s ability to handle complex computations, offering insights into the trade-offs between decentralized and centralized architectures, aiding in selecting the optimal framework for latency-sensitive applications. Figure 13 shows that processing time in the fog environment increased by 1737.67% with growing block size, indicating a near-linear relationship, while in the cloud, it rose by 1718.13%, showing a slightly steeper growth. In contrast, the proposed blockchain framework exhibited the slowest increase at 219.64%, demonstrating the lowest processing time overhead. This analysis suggests that blockchain is the most efficient for handling larger block sizes, whereas cloud storage has the longest processing time, making it unsuitable for the application scenario.

**Fig. 14:**
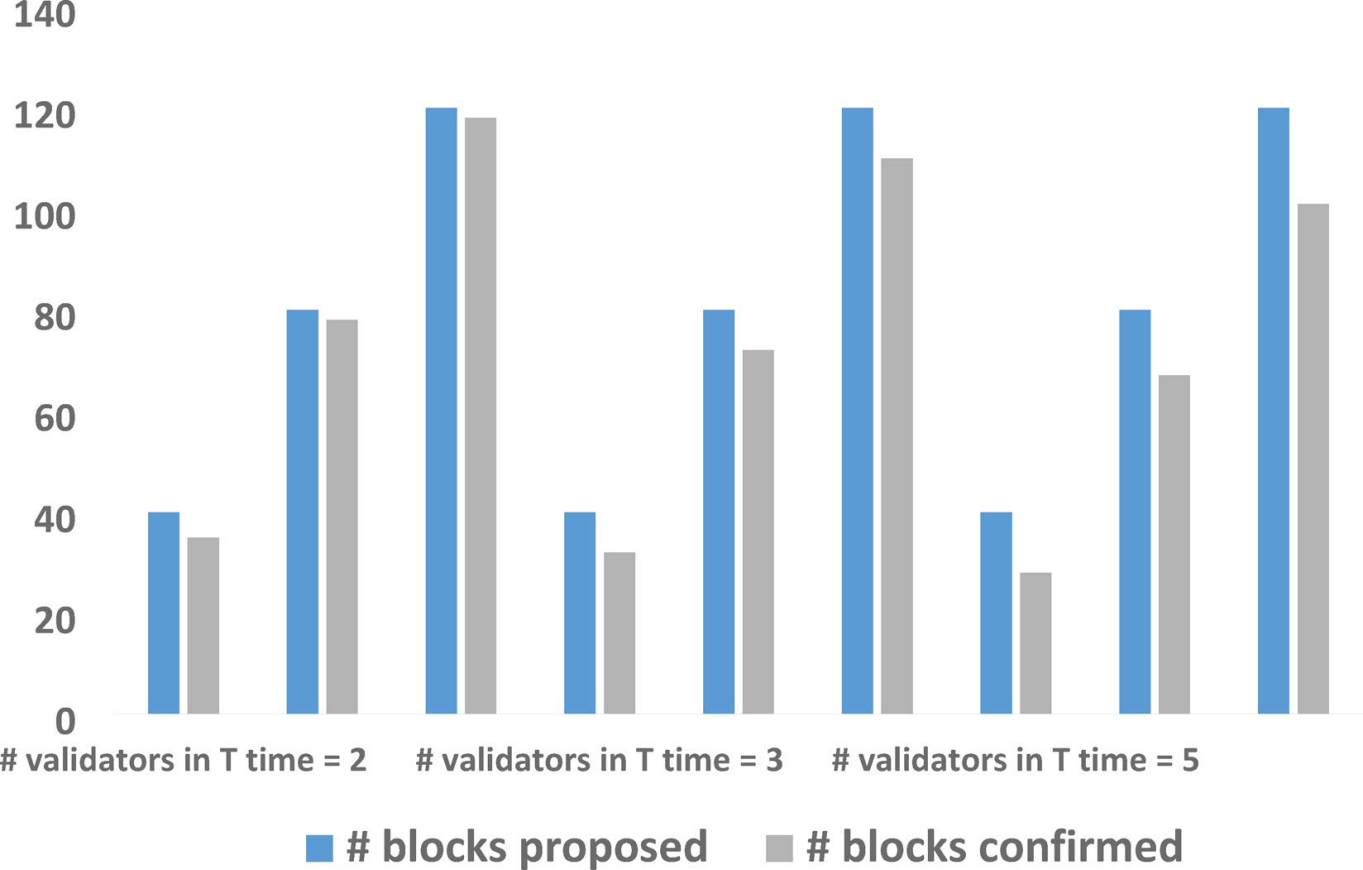
Correlation between # validators and confirmation time.

The relationship between the number of validators and blockchain confirmation time is crucial for assessing performance and scalability. The validators influence transaction validation and block creation, impacting confirmation time due to consensus mechanisms, communication overhead, and network synchronization. Analyzing this correlation balances the security, decentralization, and efficiency, offering insights into optimizing validator configurations for improved transaction throughput and reduced latency in real-world applications. In the proposed consortium blockchain framework, only pre-selected nodes dynamically validate blocks, reducing computational complexity. The relationship between validators and network performance aligns with proposed and confirmed block counts as shown in Fig. 14. The testing revealed that increasing validators introduces communication overhead, affecting transaction finality. With blocks set at 40, 80, and 120 for different dynamic times, results show that higher validator counts increase transaction latency. For instance, 100 transactions are validated in under a second with two validators, while three or five validators require more time. Given the objective of the system to validate 298 transactions every 5 s, using more than three validators is suboptimal.

**Table 11:** Comparison of communication costs (CC).

Scheme	Vehicle - sensors	Edge node	Blockchain network	Total CC time (ms)	Communication cost (bits)
Han et al.^29^	2TH + TE	TH + TY	2TY + 3TE	2.5	5120
Lu et al.^30^	TE + TY	2TH + TE	TH + TY + TE	3	4200
Liu et al.^31^	TH + TE + TY	3TE + TY	2TY + TH	4.2	5000
Kazi et al.^32^	3TH + TY	2TH + TE	3TY + TH	5.1	5300
Huang et al.^33^	2TE + TH	TH + 2TY	TE + 2TY + TH	4.8	4780
Muzumdar et al.^34^	TH + TE + TY	2TH + TE	TY + 3TE	3.3	3800
Proposed	TH + 2TE	TE + TH	2TY + TH + TE	2.1	2900

Table 11 Compares blockchain-based communication schemes in IoT systems, analyzing cryptographic operations like hashing (TH), encryption (TE), and signature operations (TY). It highlights trade-offs between computational efficiency and communication costs, showcasing The advantages of The proposed model in secure IoT communications. The schemes outline operations across vehicle sensors, edge nodes, and The blockchain network, detailing computation time (ms) and communication cost (bits). The proposed scheme outperforms others with The lowest total computation time of 2.1 Ms and a communication cost of 2900 Bits due to three technical optimizations: (i). The streamlined cryptographic operations at The vehicle-sensor layer minimize redundant steps with only TH + 2TE, rather than multiple rounds of hashing or encryption, which reduces both computational overhead and The size of transmitted data. (ii). The use of edge nodes for local preprocessing allows IoT emission data to be aggregated, validated, and encrypted at The source before transmission, reducing The volume of Raw data sent to The blockchain and thus Lowering bandwidth requirements and network congestion. (iii). The use of a consortium blockchain architecture with a limited set of pre-selected validators significantly reduces consensus complexity and inter-node communication, as fewer validators and optimized signature operations are required, minimizing network overhead.

The results demonstrate that the blockchain-based vehicle emission monitoring system outperforms fog and cloud computing in prediction accuracy, throughput, response time, and processing efficiency. XGBoost on the consortium blockchain showed up to 11% higher accuracy and 90% faster response times compared to traditional PUCC systems. The decentralized validation enhances data integrity and predictive reliability, consistently yielding better F-measure scores, especially on larger datasets. The throughput analysis reveals that while fog computing excels in local high-speed processing, the consortium blockchain scales better with increasing data volume. The latency measurements show blockchain response times comparable to fog computing and much lower than cloud systems. The processing times grow slower on blockchain, indicating better handling of larger workloads. However, increasing the number of blockchain validators raises confirmation delays, with an optimal validator count of two to three for efficient operation. The communication cost comparison shows the efficiency of the proposed scheme, enabled by streamlined cryptography, edge preprocessing, and consortium blockchain, reducing computation time and bandwidth. Hence, the blockchain framework provides a secure, scalable, and efficient alternative to traditional centralized and edge systems for real-time IoT emission data monitoring and analysis.

## Conclusion

This work presents a novel framework to enhance the PUCC system using blockchain and machine learning by integrating DApp and smart contracts to ensure secure, transparent, and automated PUCC issuance. The IoT sensors and edge nodes enable real-time, accurate vehicle emission monitoring, enhancing data reliability and decentralization. The consortium blockchain hub provides continuous vehicle tracking, transparency, and trust. The MVIs validate and digitally endorse emission records, while an incentivization mechanism encourages user participation through redeemable tokens. The XGBoost model predicts emission patterns and suggests maintenance intervals, promoting proactive engagement. The system processes 298 transactions every 5 s with a mean response time of 62.8196 ms. Notably, XGBoost on the consortium blockchain achieves 99.986% accuracy and a 99.4096% F-measure, demonstrating its improved performance. As the future work, it is planned to integrate novel blockchain consensus mechanisms while enhancing privacy-preserving techniques to improve trust. These enhancements enable the framework to serve diverse stakeholders effectively, including government, vehicle owners, environmental organizations, and manufacturers, in monitoring vehicle emissions and promoting sustainable development. These studies allow iterative refinement of our framework and validate its integration within smart city ecosystems. For real-world deployment, it is essential to ensure strict compliance with legal, regulatory, and environmental frameworks, particularly with respect to data privacy and cross-border data sharing. Accordingly, the immediate objective is to launch pilot projects with public sector partners, enhance system efficiency, and develop policy guidelines for scalable, sustainable deployment.

## Data Availability

The datasets used and/or analysed during the current study are available from the corresponding author on reasonable request.

## Code availability

The source code supporting the findings of this study is available from the corresponding author upon reasonable request. A prototype implementation is accessible at https://github.com/nikil-sri-shen/Vehicle-Emission-Monitoring-system. The data was obtained via the following UK government link:https://carfueldata.vehicle-certification-agency.gov.uk/downloads/default.aspx.
